# Polydopamine‐Based Antioxidant Countermeasures Against Spaceflight‐Induced Neurodegeneration

**DOI:** 10.1002/smsc.202500510

**Published:** 2025-12-15

**Authors:** Alessio Carmignani, Attilio Marino, Matteo Battaglini, Melike Belenli Gümüş, Elisa Carrubba, Michele Balsamo, Giovanni Valentini, Gabriele Mascetti, Marco Vukich, Giada Graziana Genchi, Gianni Ciofani

**Affiliations:** ^1^ Smart Bio‐Interfaces Istituto Italiano di Tecnologia Viale Rinaldo Piaggio 34 56025 Pontedera Italy; ^2^ Kayser Italia S.r.l. Via di Popogna 501 57128 Livorno Italy; ^3^ Agenzia Spaziale Italiana Via del Politecnico snc 00133 Roma Italy; ^4^ HESpace ESA/ESTEC Keplerlaan 1 Noordwijk 2200 AG The Netherlands; ^5^ Department of Bioscience Biotechnology and Environment University of Bari Aldo Moro Via Orabona 4 70125 Bari Italy

**Keywords:** cosmic radiation, microgravity, neuroprotection, oxidative stress, polydopamine nanoparticles

## Abstract

Exposure to microgravity and cosmic radiation during spaceflight is responsible for oxidative stress onset, contributing to neuronal dysfunction and degeneration. The central nervous system is particularly vulnerable to redox imbalance and requires effective countermeasures to ensure astronaut health and performance on long‐duration missions. In this study, the neuroprotective properties of polydopamine nanoparticles (PDNPs), known for their antioxidant activity, are investigated on neuron‐like cells exposed to different gravitational and radiation regimes. Culture conditions included administration of PDNPs and permanence aboard the International Space Station (ISS) or on a random positioning machine. Transcriptomic analyses are conducted to assess gene expression alterations associated with oxidative stress, nuclear and mitochondrial integrity, and dopamine metabolism. In‐flight, PDNP treatment mitigates the transcriptional changes induced by space stressors, preserving neuronal homeostasis. Notably, expression of key antioxidant defense genes, mitochondrial function markers and dopamine metabolism genes is stabilized in PDNP‐treated neurons. This study provides preliminary evidence on the efficacy of PDNPs in protecting neuronal cells from the combined stressors associated with spaceflight: these findings suggest PDNPs as a promising countermeasure for space‐induced neurodegeneration and support their potential translational application in the treatment of oxidative stress‐related neurodegenerative pathologies on Earth.

## Graphical abstract

Polydopamine nanoparticles (PDNPs) protect neuron‐like cells from microgravity‐ and cosmic radiation‐induced oxidative stress. Through in‐flight and ground‐based experiments, it has been shown that PDNPs preserve neuronal and mitochondrial function, markedly mitigating transcriptional dysregulation caused by spaceflight. These findings highlight PDNPs as promising neuroprotective countermeasures for long‐duration missions.

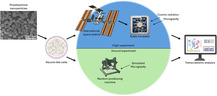

## 1 Introduction

Spaceflight exposes biological systems to a combination of microgravity (μ*g*) and cosmic radiation (CR), both of which are potent inducers of oxidative stress, a condition characterized by an imbalance between reactive oxygen species (ROS) production and antioxidant defenses, leading to biomolecular damage, inflammation, and impaired cellular function.^[^ ^1^, ^2^ ^]^ Due to its high oxygen consumption and limited endogenous antioxidant capacity, the central nervous system (CNS) is particularly vulnerable to oxidative stress‐driven injury.^[^ ^3^, ^4^ ^]^ Both μ*g* and CR have been shown to contribute to cognitive decline, neurodegeneration, synaptic alterations, and neuroinflammation, posing significant challenges to long‐duration human missions.^[^ ^5^ ^]^

Although simulated models of μ*g* (e.g., random positioning machine: RPM, rotating‐wall vessels, and hindlimb suspension) and ground‐based radiation experiments have yielded valuable insights, they fail to fully replicate the complex physiological responses observed in real spaceflight.^[^ ^6^, ^7^ ^]^ Indeed, transcriptomic and proteomic studies in animal models have revealed that gene expression changes under real μ*g* often cannot be reproduced by simulated analogs.^[^ ^8^, ^9^ ^]^ Additionally, linear energy transfer (LET) cosmic rays, such as high atomic number energy (HZE) nuclei, elicit persistent neuronal oxidative damage and dopaminergic system dysfunction even at low doses, effects not fully mirrored by gamma or proton radiation in ground models.^[^ ^10^ ^]^ A significant gap thus remains in fully understanding and distinguishing the effects of real μ*g* from those induced by simulated microgravity (sμ*g*) and CR on the CNS, underscoring the importance of validating ground‐based studies through complementary spaceflight experiments.

Distinct evidence highlights that the dopaminergic neuronal system is especially susceptible to spaceflight stress, with radiation‐linked striatal dopamine degradation and behavioral alterations reminiscent of Parkinson's disease pathophysiology.^[^ ^11^ ^]^ Emerging transcriptomic analysis confirms the convergence of space‐induced gene regulation changes with those associated with neurodegenerative disorders, reinforcing the need for protective strategies.^[^ ^12^ ^]^ Given these risks, antioxidant countermeasures are promising approaches for neuroprotection in space. Traditional small‐molecule antioxidants (e.g., vitamins, glutathione, *α*‐lipoic acid) provide only transient benefits and suffer from poor stability and distribution in vivo.^[^ ^13^ ^]^ Nanotechnology‐based antioxidants, especially those mimicking natural redox systems, instead offer enhanced stability, multi‐ROS‐scavenging capacity, and controlled delivery potential.^[^ ^14^ ^]^

Polydopamine nanoparticles (PDNPs) stand out as biodegradable, organic nanostructures with intrinsic ROS‐scavenging capability and catechol‐rich chemistry that mimics neuromelanin and supports dopamine‐like functions.^[^ ^15^ ^]^ PDNPs have demonstrated potent antioxidant and neuroprotective effects in vitro, including prevention of mitochondrial dysfunction, promotion of neurite outgrowth, and modulation of cell survival pathways in neuron‐like cells.^[^ ^16^, ^17^ ^]^ Moreover, other polydopamine‐based formulations, such as polydopamine‐coated quercetin nanoparticles, effectively mitigate neuroinflammation and neuronal apoptosis in stroke models, further confirming their neurotherapeutic potential.^[^ ^18^ ^]^ Despite these promising properties, the combined effects of μ*g* and CR on neurons, and the capacity of PDNPs to counteract space‐induced oxidative stress, have never been studied under real spaceflight conditions.

Here, we report the results of the *PROMETEO* mission aboard the International Space Station (ISS), during which neuron‐like cell cultures, either treated or not with PDNPs, were exposed to both simulated Earth gravity (s1*g*, via the centrifuge of the ESA Kubik incubator facility) and real μ*g*, simultaneously experiencing CR by permanence in low Earth orbit. Following return to Earth, transcriptomic profiling was performed to analyze the regulation of genes linked to antioxidant defense, mitochondrial integrity, and dopamine metabolism. A control experiment was also conducted on ground for assessment of shielding from CR, while cultures were either exposed to sμ*g* or Earth gravity (1*g*). By comparing treated vs. untreated conditions across gravity states, this work aims at identifying the distinctive molecular impact of μ*g* and CR, and to assess PDNP‐mediated neuroprotection under authentic spaceflight stressors. To our knowledge, this represents the first transcriptomic investigation evaluating a biodegradable antioxidant nanomaterial on neuronal cells in real spaceflight. Our findings may have implications not only for astronaut health during long‐term missions, but also for developing therapeutic avenues in Parkinson's disease and other oxidative stress‐related CNS disorders on Earth.

## 2 Results

### 2.1 Nanoparticle Characterization

**Figure 1:**
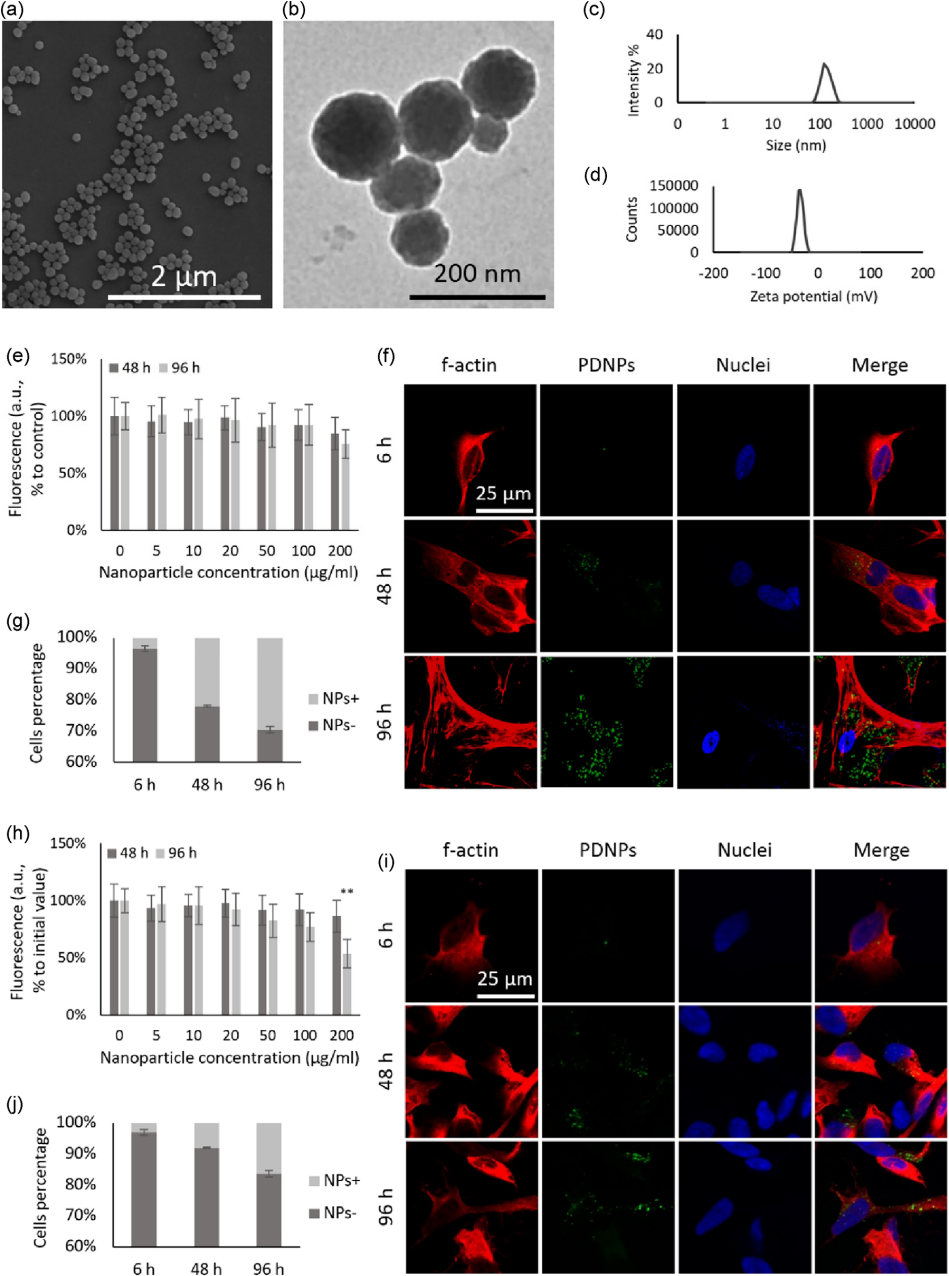
Material characterization and cellular interactions of PDNPs. Representative a) SEM and b) TEM images. c) Hydrodynamic diameter distribution and d) *ζ*‐potential analysis. e) PicoGreen assay, indicative of cell viability, in Earth gravity, and assessment of PDNP internalization in the same regimen: f) representative confocal microscopy images (f‐actin in red, DiO‐PDNPs in green, nuclei in blue) and g) flow cytometry analysis (representative plots in Figure S2, Supporting Information). h) PicoGreen assay in simulated microgravity (**p < 0.01), and assessment of PDNP internalization in the same regimen: i) representative confocal microscopy images (f‐actin in red, DiO‐PDNPs in green, nuclei in blue) and j) flow cytometry analysis (representative plots in Figure S3, Supporting Information).

Scanning electron microscopy (SEM; **Figure** **1**a) and transmission electron microscopy (TEM; Figure 1b) analysis revealed that the nanoparticles exhibited a spherical morphology, with an average diameter of 102.5 ± 12.6 nm. Dynamic light scattering (DLS) measurements confirmed a narrow size distribution, reporting a hydrodynamic diameter (*D*_h_) of 126.8 ± 13.4 nm and a polydispersity index (PDI) of 0.07 ± 0.09 (Figure 1c). Additionally, the particles displayed a *ζ*‐potential of −33.7 ± 0.9 mV (Figure 1d).

Nanoparticle stability in biological environments was confirmed by dispersing PDNPs in differentiation medium (for the composition please check the Experimental Section). Throughout the observation period, no significant changes were observed in *D*_h_ or PDI, suggesting nanoparticles possess high stability under physiological conditions (Figure S1, Supporting Information).

The antioxidant capacity of PDNPs was evaluated in terms of Trolox equivalents, a standard reference antioxidant. The results indicated that 1 μg ml^−1^ of PDNPs corresponds to 69.77 ± 0.60 μM of Trolox, confirming their intrinsic antioxidant activity. To benchmark this value, we performed the same assay with 100 μM ascorbic acid, 10 μM tannic acid, and 100 μM idebenone, obtaining, respectively, 78.25 ± 1.09, 106.13 ± 0.94, and 134.67 ± 2.11 μM of Trolox equivalent activity (Table S1, Supporting Information).

### 2.2 Evaluation of Nanoparticle‐Cell Interactions

To assess the biological interactions between PDNPs and neuron‐like cells, cellular viability was first evaluated. Cells were treated with increasing concentrations of PDNPs (0 to 200 μg mL^−1^), and viability was measured after 48 h and 96 h by using a fluorimetry assay, which quantifies dsDNA content as an indicator for cell viability (Figure 1e). Across all tested conditions, no statistically significant reduction in cell viability was observed (*p* > 0.05). We selected 20 μg mL^−1^ as working concentration for the following experiments, a dose that, based on previous experiences, was found not affecting the activation mechanisms of the experimental units (EUs) reservoirs.

Internalization of DiO‐labeled PDNPs (20 μg mL^−1^) was assessed by using confocal microscopy and flow cytometry, both of which showed a time‐dependent uptake. Specifically, 3.5 ± 0.9% of cells internalized the nanoparticles after 6 h, 21.9 ± 0.2% after 48 h, increasing to 29.4 ± 1.0% after 96 h (Figure 1f,g, and S2, Supporting Information).

The same set of experiments was conducted under sμ*g* conditions by using an RPM. Under these conditions, PDNP exposure did not affect cell viability after 48 h (*p* > 0.05). However, a statistically significant decrease in viability (*p* < 0.01) was observed after 96 h at the highest concentration tested (200 μg mL^−1^, Figure 1h).

Time‐dependent cellular uptake of DiO‐PDNPs (20 μg mL^−1^) under sμ*g* was also confirmed by both confocal microscopy and flow cytometry, with internalization increasing from 3.0 ± 0.8% at 6 h, to 8.1 ± 0.1% at 48 h, and to 16.4 ± 0.2% at 96 h, similar to the pattern observed in 1*g* conditions (Figure 1i,j, and S3, Supporting Information).

To confirm the antioxidant properties of polydopamine‐based nanostructures, the ability of PDNPs to modulate intracellular reduced glutathione (GSH) levels was quantitatively examined. In 1*g* conditions (Figure S4a, Supporting Information), control cells contained 5.89 ± 0.15 ng of GSH, while PDNP‐treated cells had higher levels (6.94 ± 0.16 ng). Following exposure to a pro‐oxidant stimulus (tert‐butyl hydroperoxide ‐TBH‐), GSH levels decreased to 4.03 ± 0.10 ng in untreated cells, whereas PDNP‐treated cells maintained higher levels at 6.54 ± 0.17 ng (*p* < 0.001). In sμ*g* conditions (Figure S4b, Supporting Information), baseline GSH levels were 4.98 ± 0.29 ng in control cells and 5.02 ± 0.27 ng in PDNP‐treated cells. Upon oxidative insult, GSH levels in control cells decreased to 3.01 ± 0.34 ng, while PDNP ± treatment helped to preserve higher levels (4.17 ± 0.35 ng, *p* < 0.001).

The antioxidant properties of PDNPs were also studied by evaluating the intracellular levels of oxidative stress. In 1*g* conditions (Figure S5a,b, Supporting Information), treatment with TBH increased cytosolic ROS levels, with the percentage of ROS‐positive cells rising from 6.3 ± 0.3% in control to 38.6 ± 1.2% after exposure to TBH. PDNP treatments, compared to control cells, slightly reduced the basal level of oxidative stress by lowering the ROS‐positive cells to 5.8 ± 0.2%. On the other hand, in the presence of TBH, we observed a significant lowering in ROS‐positive cells to 15.8 ± 0.4%, due to PDNP treatment (*p* < 0.001). In sμ*g* conditions (Figure S5c,d, Supporting Information), the basal level of ROS‐positive cells increased to 17.7 ± 2.1%, and to 41.6 ± 3.8% in the presence of TBH. PDNP treatments reduced the basal level of ROS by lowering the ROS‐positive cells to 6.4 ± 0.6%, while after TBH exposure, we observed a significant lowering in ROS‐positive cells to 27.0 ± 1.4% (*p* < 0.001).

The protective effects of PDNPs were also evaluated in mitochondria. In 1*g* conditions (Figure S6a,b, Supporting Information), TBH treatment increased the number of cells positive for mitochondrial ROS, from 8.9 ± 0.3% in control to 15.2 ± 0.7% in the presence of TBH. PDNPs reduced mitochondrial ROS levels, with ROS‐positive cells slightly decreasing to 8.0 ± 0.8% in control conditions and significantly reducing to 10.0 ± 0.3% after TBH exposure (*p* < 0.001). In sμ*g* conditions (Figure S6c,d, Supporting Information), the same trend was observed, with TBH treatment increasing the number of cells positive for mitochondrial ROS from 9.4 ± 0.3% in control to 20.2 ± 1.3% in the presence of TBH, while PDNPs reducing mitochondrial ROS levels to 8.3 ± 0.1% in control conditions, and significantly reducing to 13.2 ± 0.5% after TBH exposure (*p* < 0.001). Overall, the mitochondrial membrane potential appeared to remain stable, as neither the pro‐oxidant challenge nor the alteration in gravitational load produced any appreciable shift in fluorescence intensity across the different experimental groups (Figure S7, Supporting Information).

### 2.3 Influence on Neuronal Differentiation and Neurite Outgrowth

**Figure 2:**
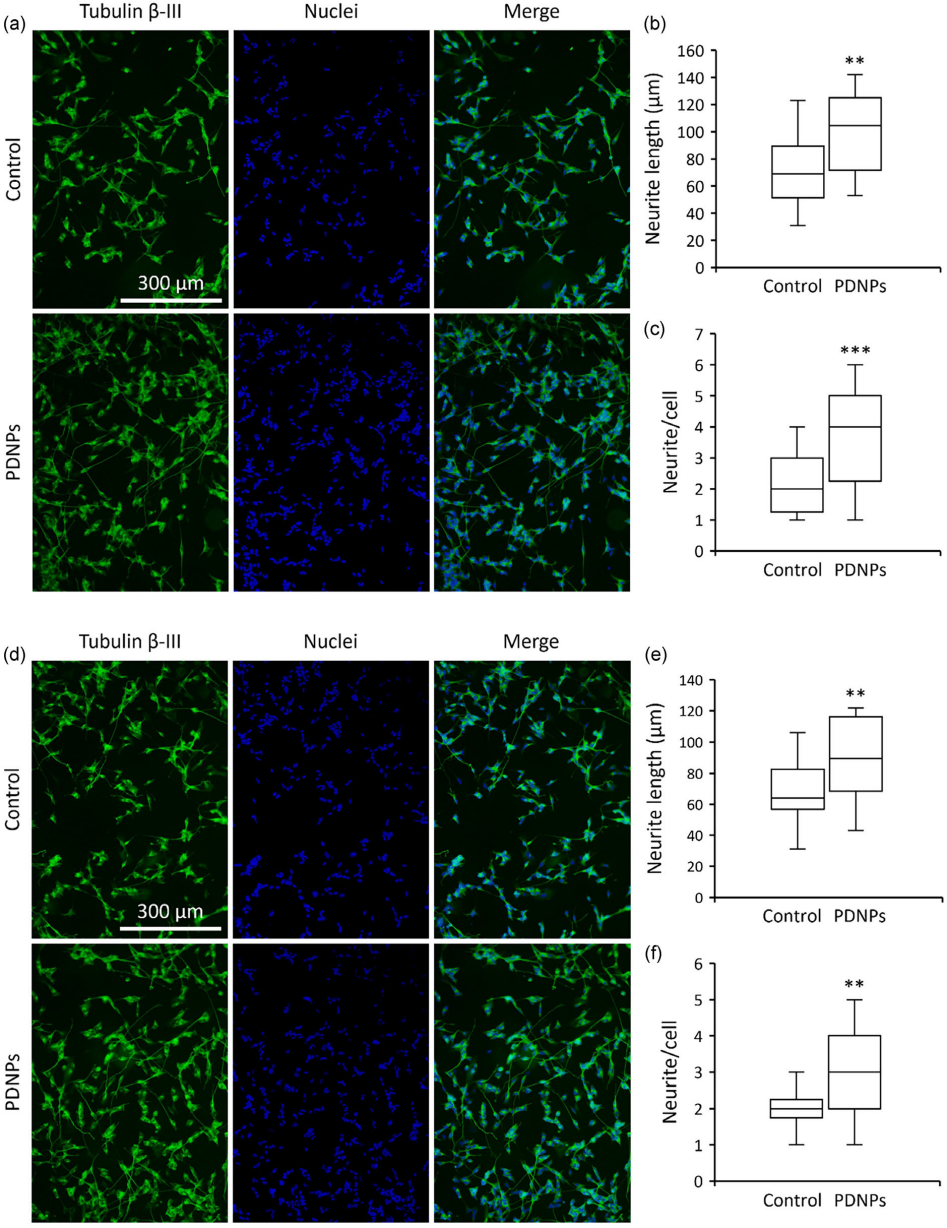
Analysis of PDNP effect on neurite outgrowth. a) Representative epifluorescence images (tubulin β‐III in green, nuclei in blue), b) median neurite length (** *p* < 0.01), and c) neurite/cell ratio (*** *p* < 0.001) for cultures in Earth gravity regimen. d) Representative epifluorescence images (tubulin β‐III in green, nuclei in blue), e) median neurite length (** *p* < 0.01), and f) neurite/cell ratio (*** *p* < 0.001) for cultures in simulated microgravity.

The potential of PDNPs to enhance neuronal differentiation and neurite extension was assessed by using epifluorescence microscopy (**Figure** **2**a). After 96 h in differentiation medium, neuron‐like cells exhibited a median neurite length of 69.0 ± 1.6 μm. When the medium was supplemented with 20 μg mL^−1^ of PDNPs, neurite length significantly increased to 104.5 ± 1.4 μm (*p* < 0.01; Figure 2b). Similarly, the median number of neurites *per* cell rose from 2.00 ± 0.17 in untreated cells to 4.00 ± 0.05 in PDNP‐treated cells (*p* < 0.001; Figure 2c).

These results were consistent in sμ*g* conditions (Figure 2d). In absence of nanoparticles, cells displayed a median neurite length of 64.0 ± 1.1 μm and 2.00 ± 0.05 neurites per cell (*p* < 0.01; Figure 2e). Treatment with PDNPs under the same conditions led to a significant increase in both metrics, with neurite length reaching 89.5 ± 0.98 μm and neurite count rising to 3.00 ± 0.04 (*p* < 0.01; Figure 2f).

### 2.4 In‐Flight Experiments

**Figure 3:**
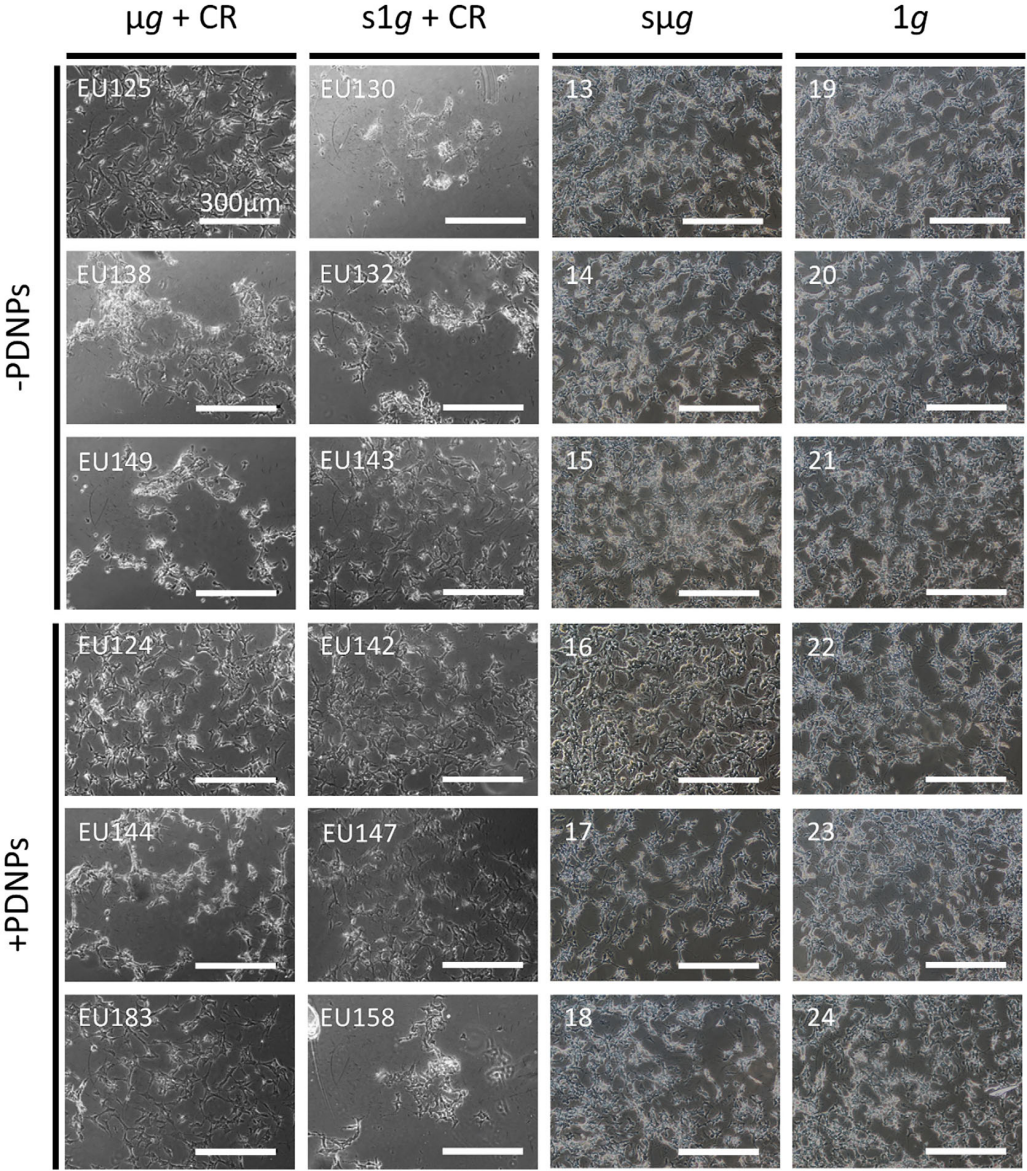
Representative phase‐contrast optical microscopy images of neuron‐like cells at experiment endpoint. (μ*g* = microgravity, CR = cosmic radiation, s1*g* = simulated Earth gravity, sμ*g* = simulated microgravity, 1*g* = Earth gravity, EU = experimental unit).

As shown in Figure S8, Supporting Information, cell cultures experienced a gradual temperature decrease from +30 °C to +28 °C prior to insertion of the experiment containers (ECs), enclosing the EUs for automated conduction of the cell cultures in‐flight, into the Kubik incubator on board the ISS at 73 h from payload launch (L + 73 h, where “L” indicates the NG‐18 Antares rocket launch time). At the end of the experimental timeline, cultures remained viable and adherent (**Figure** **3**). The spaceflight experiment was successfully replicated on Earth, with sμ*g* achieved with 3D clinorotation on a RPM. In this case as well, viable and adherent cultures were observed after the experiment (Figure 3).

### 2.5 RNA Extraction and Next‐Generation Sequencing

Following RNA extraction, purification, and quality assessment (Figure S9, Supporting Information), transcriptomic analysis was conducted to identify gene pathways affected by μ*g*, CR, and to assess the effects of PDNP treatment. The number of differentially expressed genes (DEGs) for the nine selected pairwise comparisons among experimental groups is reported in Table S2, Supporting Information. In neuron‐like cells, PDNP treatment alone (H vs. G) resulted in the differential expression of 332 genes, whereas PDNP treatment under spaceflight conditions (B vs. A) led to 1,654 DEGs. Assessment of the rescue effects of PDNPs under spaceflight conditions (B vs. G) revealed the differential expression of a total of 487 genes. Exposure to combined μ*g* and CR (A vs. G) resulted in 1,472 DEGs, while μ*g* alone (A vs. C) yielded 2,838 DEGs, and CR alone (C vs. G) 3,702 DEGs. PDNP treatment in sμ*g* (F vs. E) produced a differential expression of 1,657 genes, whereas the evaluation of PDNP rescue effects under μ*g* (B vs. C) and CR (B vs. E) conditions revealed 593 and 2,144 DEGs, respectively.

**Figure 4:**
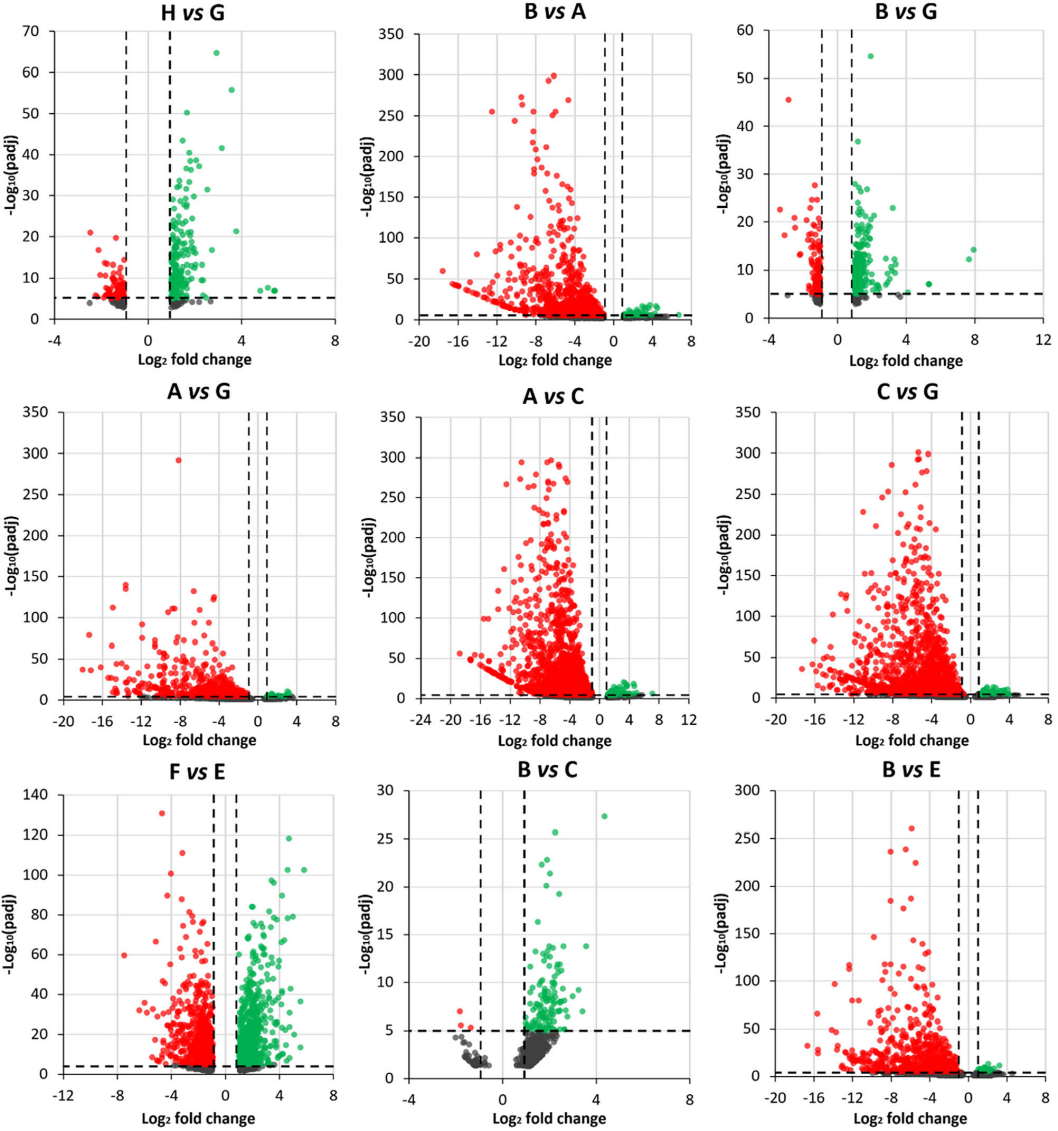
Scatter plots for the nine selected comparisons among the experimental classes sequenced successfully. Each volcano plot shows significance (*y*‐axis, as log_10_ of the adjusted *p*‐value, padj) and overexpression change (*x*‐axis, as log_2_ fold change) for all genes (each represented by a dot) in a given comparison. Significantly up‐ or downregulated genes are reported in green or red, respectively, while the remaining genes are indicated in black. Experimental classes: A (‐PDNP, μ*g*, +CR); B (+PDNP, μ*g*, +CR); C (‐PDNP, s1*g*, +CR); D (+PDNP, s1*g*, +CR); E (‐PDNP, sμ*g*, ‐CR); F (+PDNP, sμ*g*, ‐CR); G (‐PDNP, 1*g*, ‐CR); H (+PDNP, 1*g*, ‐CR).

**Figure 5:**
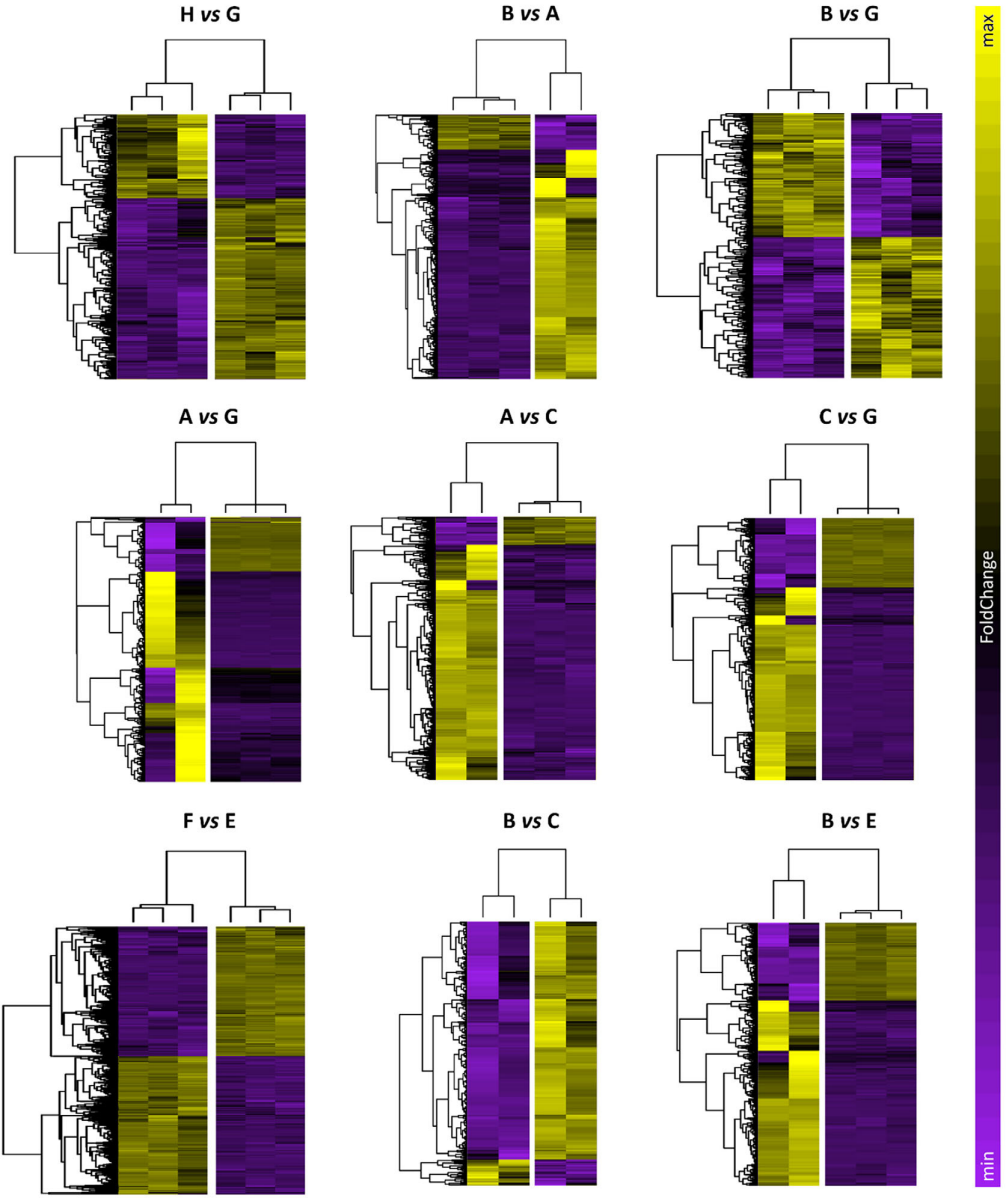
Heatmaps for the nine selected comparisons. Expression values for such genes are reported as colored bars. The hotter the color, the higher the fold change (FC; most overexpressed genes within each map are indicated in yellow, and most underexpressed genes are shown in purple). Experimental classes: A (‐PDNP, μ*g*, +CR); B (+PDNP, μ*g*, +CR); C (‐PDNP, s1*g*, +CR); D (+PDNP, s1*g*, +CR); E (‐PDNP, sμ*g*, ‐CR); F (+PDNP, sμ*g*, ‐CR); G (‐PDNP, 1*g*, ‐CR); H (+PDNP, 1*g*, ‐CR).

**Figure 6:**
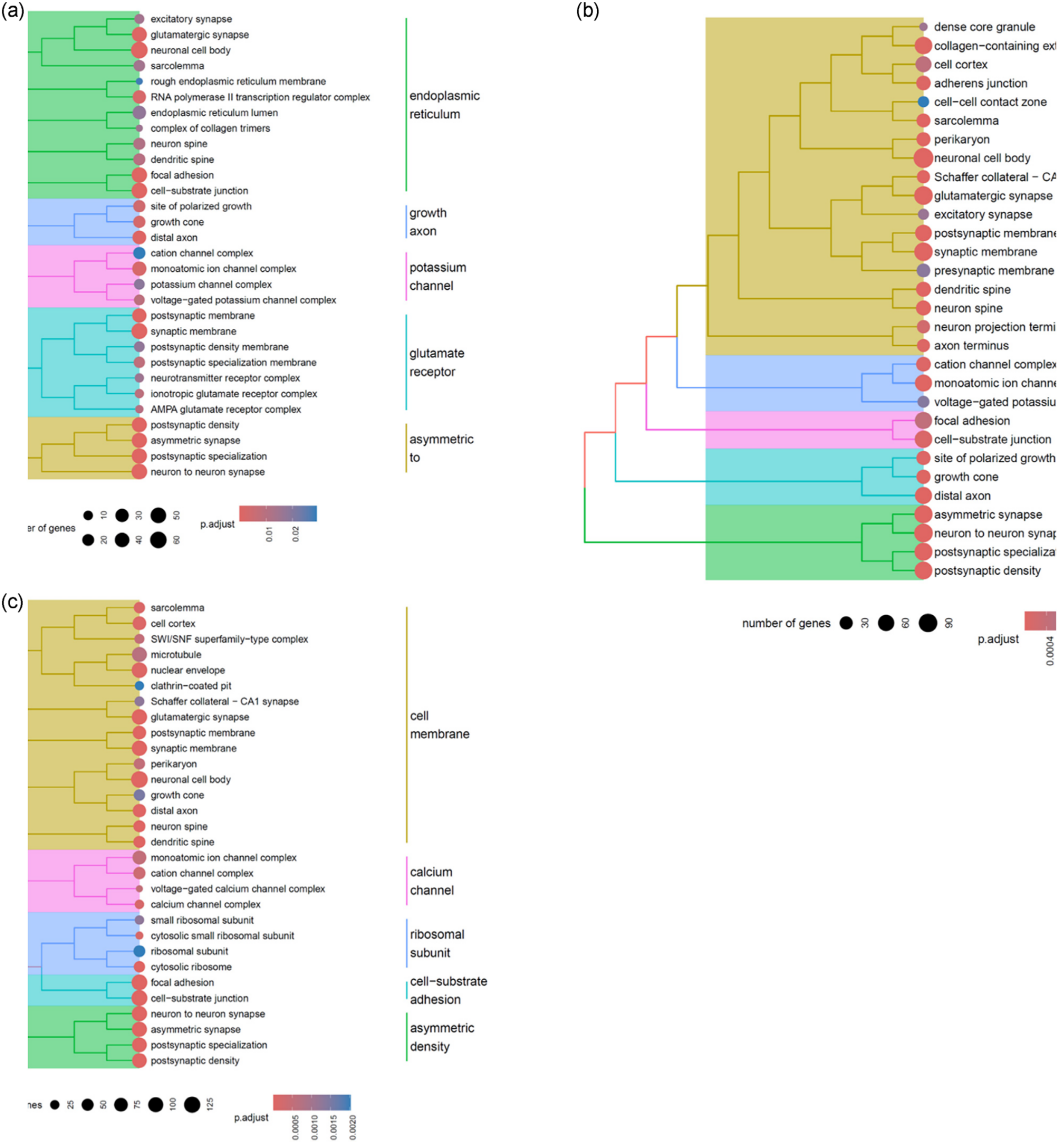
Gene ontology (GO) enrichment analysis of neuron‐like cells exposed to space‐related stressors. a) Combined effect of μ*g* and CR, b) effect of μ*g* alone, and c) effect of CR alone.

**Figure 7:**
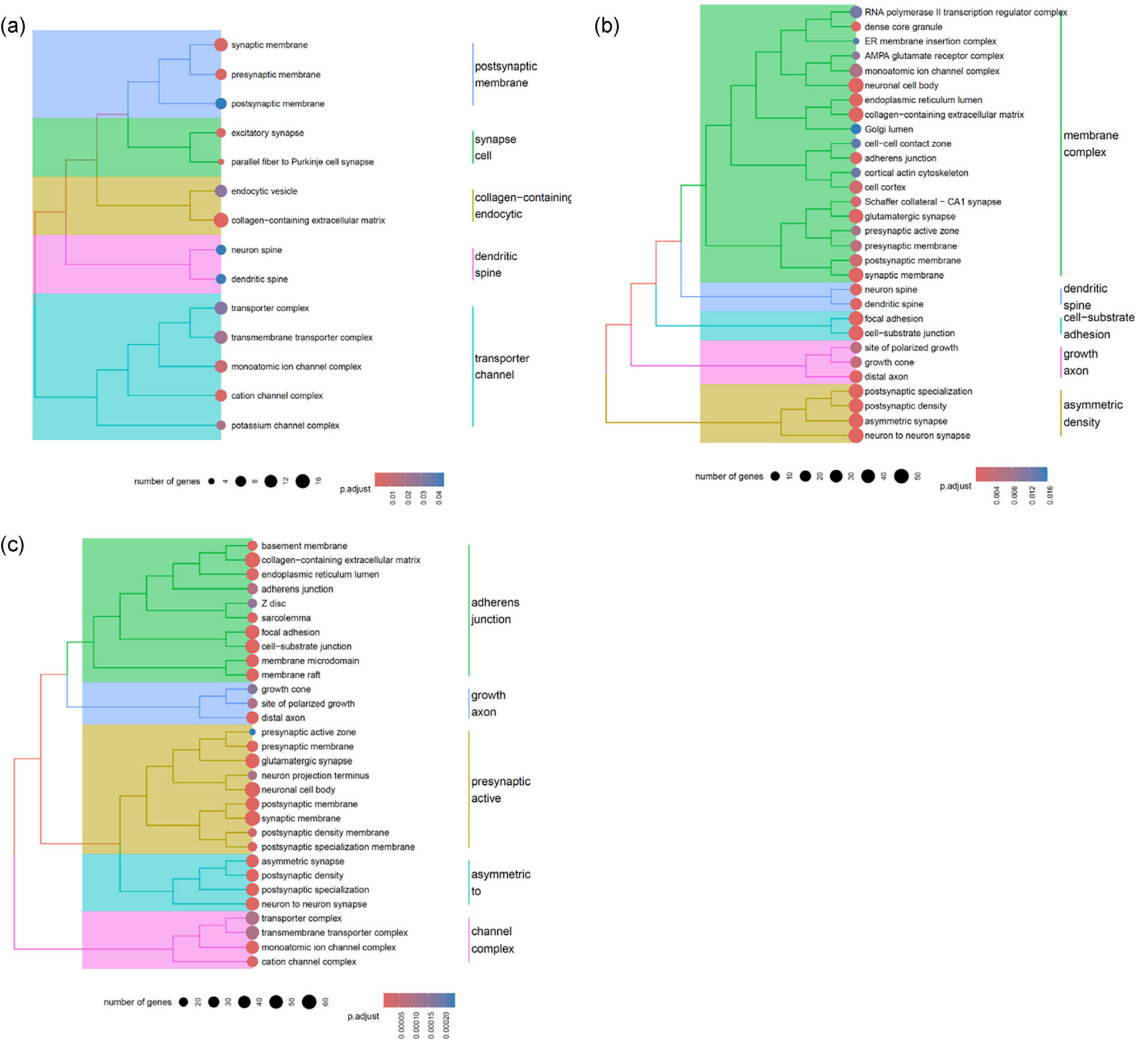
GO enrichment analysis of neuron‐like cells treated with PDNPs. a) Effect of PDNPs at 1*g*, b) protective effect of PDNPs against combined μ*g* and CR, and c) protective effect of PDNPs against sμ*g*.

**Figure 8:**
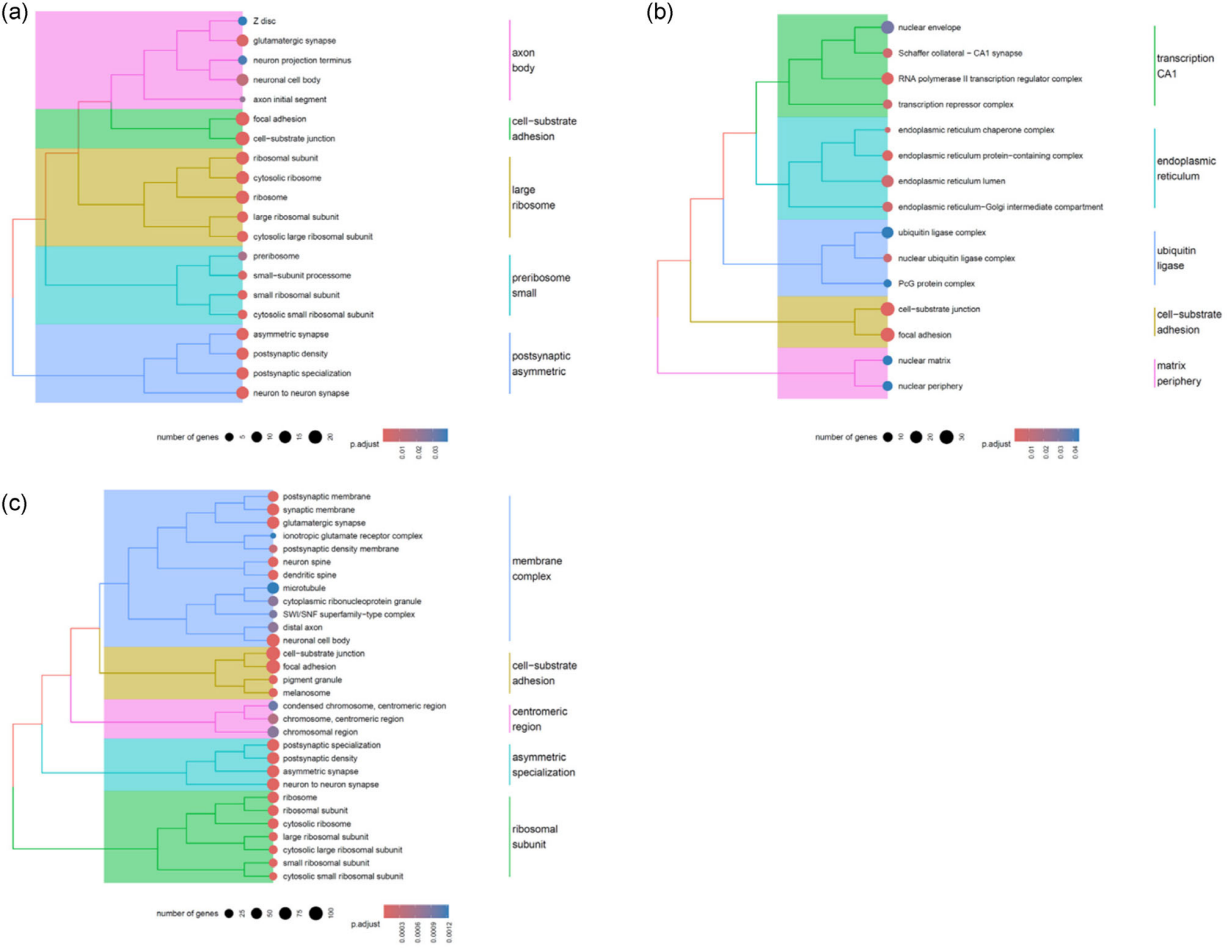
GO enrichment analysis illustrating the rescue effects of PDNPS on neuron‐like cells exposed to spaceflight‐related stressors. a) Rescue effect against combined μ*g* and CR, b) rescue effect against μ*g*, and c) rescue effect against CR.

Volcano plots, heatmaps, and gene ontology (GO) enrichment analyses are presented in **Figure** 4, 5, 6, 7, 8, and in Figure S10, Supporting Information, respectively.

## 3 Discussion

Few in vitro and in vivo studies have examined the effects of the space environment on the CNS by simulating both gravitational unloading and exposure to CR. Among these, Pani et al. exposed mouse cortical neurons to chronic ionizing radiation under sμ*g*, observing a synergistic deleterious impact on neuronal morphology, viability, and the transcription of genes involved in synaptic transmission.^[^ ^19^ ^]^ Conversely, Kulikova et al. reported that one month of sμ*g*, induced in rats through hindlimb suspension, did not alter the transcription of genes associated with long‐term spaceflight risk.^[^ ^20^ ^]^ However, a subsequent study by the same group, conducted aboard the Bion‐M biosatellite, revealed that real μ*g* caused pronounced transcriptional perturbations in these same risk genes in mice.^[^ ^21^ ^]^ Collectively, these and other studies highlight the critical need for experiments that simultaneously expose neural models to both real μ*g* and CR conditions that can only be achieved during spaceflight. Such investigations are essential to complement ground‐based studies and to design effective countermeasures against motor and cognitive impairments linked to oxidative stress‐associated neurodegeneration in space. In an attempt to fill the gap of knowledge concerning synergistic effects of μ*g* and CR, this study proposed for the first time the use of fully organic, biocompatible, and biodegradable nanoparticles as a neuroprotective agent to neurons exposed to the spaceflight environment.

In line with previous studies conducted by our group,^[^ ^22^ ^]^ the synthesized PDNPs exhibited a spherical morphology, an average diameter close to 100 nm, a monodisperse size distribution, and a strongly negative *ζ*‐potential. These nanostructures demonstrated long‐term colloidal stability in the differentiation medium supplied to the neuron‐like cells, resulting to be compatible with typical timeline of payload delivery to the ISS.^[^ ^23^ ^]^ In addition, the strong antioxidant potential was confirmed by comparing their free radical scavenging activity with that of Trolox, a water‐soluble analog of vitamin E, highlighting their capacity to neutralize ROS, a critical property for mitigating the oxidative stress typically associated with spaceflight conditions.^[^ ^24^ ^]^

To explore the interaction between PDNPs and neuron‐like cells, we first evaluated their impact on cellular viability. As aforementioned, PDNPs exhibited excellent biocompatibility and underwent a progressive, time‐dependent cellular uptake. Under sμ*g* conditions, as expected, nanoparticle internalization was reduced.^[^ ^25^ ^]^ Nevertheless, PDNPs effectively mitigated both cytosolic and mitochondrial oxidative stress levels following a pro‐oxidative stimulus, though with slightly reduced efficacy under sμ*g* with respect to 1*g*. This difference is likely attributable to the lower levels of nanoparticle internalization observed in μ*g*‐exposed cells.

The potential of PDNPs to promote neuronal differentiation was also investigated. Treatment with PDNPs significantly enhanced differentiation, as evidenced by an approximate doubling in both the median neurite length and the number of neurites per cell. This effect was also observed under sμ*g*, although to a lesser extent, again likely due to differences in internalization efficiency between the two gravity conditions. Given the key role of oxidative stress in modulating neuronal signaling pathways, such as MAPK/ERK and NGF, an environment characterized by elevated ROS can impair neurite extension.^[^ ^26^ ^]^ By attenuating oxidative stress, PDNPs establish a more favorable redox milieu that supports and promotes neuronal differentiation. These findings suggest that PDNPs may hold promise for applications in neuronal regeneration.

Furthermore, the antioxidant activity of PDNPs may help reduce the oxidative degradation of intracellular molecules such as dopamine. Previous work from our group demonstrated that PDNP treatment led to a significant increase in dopamine levels in neuron‐like cell cultures.^[^ ^27^ ^]^ This enhancement may be due not only to the ROS‐scavenging properties of polydopamine, but also to its structural and chemical resemblance to endogenous dopamine, which could influence intracellular dopamine metabolism.^[^ ^28^ ^]^ Further investigations are warranted to clarify the underlying molecular mechanisms and to explore whether this phenomenon can be exploited for neuroprotective or neuromodulatory strategies.

To further explore the neuroprotective potential of PDNPs under spaceflight conditions, we performed an in‐depth transcriptomic analysis. This strategy enabled us to investigate how PDNP treatment may modulate cellular pathways at the molecular level during concurrent exposure to μ*g* and CR. For in‐flight samples, transcriptome sequencing demonstrated alignment with two species: *Homo sapiens* and *Mycoplasma hyorhinis*, from microbial contamination of unknown origin. We applied a conservative filtering strategy, by excluding from downstream analyses the set of genes identified by Park et al. as being most consistently influenced by Mycoplasma infection. This approach aimed at minimizing artifactual transcriptional signals attributable to contamination rather than to the experimental condition under investigation.^[^ ^29^ ^]^ In the following, data are discussed in view of their relevance to oxidative stress mitigation and neuroprotection.

The analysis of the effects of μ*g* alone (comparison A vs. C) revealed a significant enrichment of numerous GO terms related to neuronal function, including *excitatory synapse*, *postsynaptic density*, and *glutamate receptor complex*. This enrichment was found upon observation of the downregulation of a large set of genes with key roles in neuronal function. Among these downregulated markers, there is *SHANK3* with a log_2_ fold change of −3.8, which encodes for a scaffold protein of the postsynaptic density, a specialized structure on the postsynaptic membrane of excitatory synapses.^[^ ^30^ ^]^ Decreased *SHANK3* expression was documented to be associated to a decrease in both the number and strength of excitatory synapses, ultimately impairing synaptic transmission and plasticity.^[^ ^31^ ^]^ Another downregulated marker is *SLC17A7* (log_2_ fold change −5.9), also known as *VGLUT1*, which encodes for a vesicular glutamate transporter. Its downregulation can decrease the vesicular content of glutamate, resulting in impaired excitatory transmission.^[^ ^32^ ^]^ Knockdown of this gene has been associated with severe synaptic defects and postnatal lethality.^[^ ^33^ ^]^ Downregulation was also observed in genes involved in mitochondrial function. Of these, *MBP*, which showed a log_2_ fold change of −2.3, encodes for a pore‐forming subunit of the mitochondrial calcium uniporter, the primary channel for mitochondrial Ca^2+^ uptake.^[^ ^34^ ^]^ Decreased expression was shown to compromise the energetic support required during periods of high neuronal firing.^[^ ^35^ ^]^ Similarly, *NDUFAF5*, with a log_2_ fold change of −3.2, encodes for an assembly factor for Complex I of the mitochondrial respiratory chain.^[^ ^36^ ^]^ Its downregulation can impair Complex I assembly, leading to neuronal energy deficits that can result in mitochondrial encephalopathy.^[^ ^37^ ^]^ Furthermore, our results suggest that μ*g* may disrupt dopamine metabolism. The *SLC18A1* gene (log_2_ fold change −3.0) encodes for the vesicular monoamine transporter VMAT1, responsible for the packaging of monoamines (including dopamine) into synaptic vesicles.^[^ ^38^ ^]^ In murine models, downregulation of this gene has been shown to impair dopaminergic signaling.^[^ ^39^ ^]^

Similarly, in the case of exposure to CR (comparison C vs. G), we observed the enrichment of multiple GO terms associated with synaptic activity, such as s*ynaptic membrane, neuron‐to‐neuron synapse,* and *dendritic spine,* again depending on the downregulation of several genes indicative of neuronal impairment. Of these, with a log_2_ fold change of −1.5, the *RELN* gene encodes for the extracellular glycoprotein reelin, which plays a pivotal role in neuronal migration and cortical lamination during development, as well as in synaptic maturation and transmission in adulthood.^[^ ^40^ ^]^ Reduced reelin expression in humans has been associated with severe neuronal migration deficits, such as those reported in the cortex of patients with schizophrenia.^[^ ^41^ ^]^ In our study, *NLGN1* (log_2_ fold change −1.9) is another marker that underwent downregulation, which typically leads to weakened excitatory synaptic transmission and impaired long‐term potentiation, thereby compromising synaptic plasticity.^[^ ^42^ ^]^ This effect is attributable to the role of *NLGN1* in encoding neuroligin‐1, a postsynaptic adhesion molecule specifically involved in the formation and maintenance of excitatory synapses.^[^ ^43^ ^]^ CR exposure was also found to interfere with mitochondrial activity. Among the downregulated markers, with a log_2_ fold change of −2.3, there is also *SLC25A28*, which encodes for a member of the mitochondrial solute carrier family SLC25, responsible for transporting iron into the mitochondrial matrix, which is essential for the biosynthesis of heme groups and iron–sulfur clusters.^[^ ^44^ ^]^ Decreased expression of *SLC25A28* leads to reduced mitochondrial iron import, resulting in impaired respiratory chain function and, consequently, increased oxidative stress.^[^ ^45^ ^]^ We also observed interference with the dopaminergic system. Specifically, lower expression of *GNAL* (log_2_ fold change −3.7), which encodes for a protein involved in dopamine D1‐D5 receptor signal transduction through the cAMP pathway, may lead to striatal dopaminergic signaling deficits.^[^ ^46^ ^]^ Loss‐of‐function mutations in *GNAL* are known to cause primary dystonia in humans, consistent with hypofunction of dopaminergic circuits.^[^ ^47^ ^]^ Additionally, we found downregulation of *LMX1B*, which showed a log_2_ fold change of −7.1, responsible for the production of a transcription factor required for the specification and maintenance of midbrain dopaminergic neurons.^[^ ^48^ ^]^ Together with *LMX1A*, these two genes activate key developmental and quality‐control programs essential for dopaminergic neuronal identity and function.^[^ ^49^ ^]^

The study of the synergistic effects of μ*g* and CR (comparison A vs. G) highlighted the enrichment of GO terms previously identified, including *glutamatergic synapse, postsynaptic specialization,* and *focal adhesion*, arising from the downregulation of many genes already observed in the previous comparisons on the effects of μ*g* or CR alone. Of particular interest, we identified several genes that were consistently downregulated across all three conditions, namely when μ*g* or CR was considered individually, as well as when they acted in combination. One example, with a log_2_ fold change of −6.6, is *CAMK2B*, encoding for the β‐subunit of calcium/calmodulin‐dependent protein kinase II, a key regulator of synaptic plasticity through modulation of actin cytoskeleton architecture.^[^ ^50^ ^]^ Some studies in animal models have shown that reduced expression of this gene leads to hippocampal dysfunction.^[^ ^51^ ^]^ Another notable marker that we found to be downregulated is *MBP* (log_2_ fold change −9.9), encoding for myelin basic protein, which is essential for myelin compaction and conduction velocity.^[^ ^52^ ^]^ Decreased production of MBP protein was found to delay oligodendrocyte maturation, thereby compromising myelination supportts.^[^ ^53^ ^]^ Across all three comparisons, we also found downregulated genes associated with mitochondrial activity. For instance, *MICU3* (log_2_ fold change −2.5), a neuron‐specific regulator of mitochondrial Ca^2+^ uptake during synaptic activity, was reduced, thereby limiting the amplitude of Ca^2+^ transients and ultimately impairing synaptic function.^[^ ^54^ ^]^ Similarly, with a log_2_ fold change of −2.3, *SLC8A3*, encoding for the Na^+^/Ca^2+^ exchanger NCX3, regulates mitochondrial Ca^2+^ homeostasis, and its decreased expression negatively impacts mitochondrial performance.^[^ ^55^ ^]^ As aforementioned, dopaminergic metabolism also emerged as a potential target of the combined action of μ*g* and CR, with several genes consistently downregulated across all comparisons. Among them, there are *DHFR* (log_2_ fold change −9.8), encoding for a cofactor required for dopamine synthesis, and *PTPRN* (log_2_ fold change −1.9), a protein involved in vesicular filling and the release of hormones and neurotransmitters, including dopamine.^[^ ^56^, ^57^ ^]^

Despite the limitations of this study due to the microbial contaminations of the in‐flight cultures, these findings collectively suggest that both μ*g* and CR, individually and synergistically, may converge on common molecular pathways, leading to impaired synaptic function, disrupted mitochondrial homeostasis, and altered dopaminergic signaling, of potential threat to neuronal integrity in the space environment.

The antioxidant activity of PDNPs was first evaluated in control cells, namely those maintained under 1*g* and not exposed to CR (comparison H vs. G). This comparative approach revealed an enrichment of additional GO terms associated with neuronal activity, such as *neuron spine, endocytic vesicle,* and *cation channel complex.* Treatment with PDNPs indeed induced the differential expression of a large set of genes, including several markers with well‐established antioxidant functions. For instance, with a log_2_ fold change of 1.2, we observed the upregulation of *FOXO1*, a key transcription factor protecting cells against oxidative stress through the induction of antioxidant enzymes, such as superoxide dismutase and catalase.^[^ ^58^ ^]^ Its overexpression reduces ROS levels, preserves mitochondrial membrane integrity, and limits apoptosis.^[^ ^59^ ^]^ Another notable example is CLU, which showed a log_2_ fold change of 1.2, encoding for clusterin, a protein that acts as a biosensor for oxidative stress, particularly against peroxides.^[^ ^60^ ^]^ In *Drosophila* neuronal models, *CLU* overexpression was shown to protect neurons from proteotoxicity by reducing endoplasmic reticulum stress and the formation of toxic aggregate of proteins.^[^ ^61^ ^]^ *GPNMB*, which showed a log_2_ fold change of 1.1, is also of interest, encoding for a transmembrane glycoprotein, the overexpression of which in murine models of spinal cord injury attenuates inflammation and promotes autophagy, ultimately supporting neuronal recovery.^[^ ^62^ ^]^

Besides the antioxidant activity of PDNPs under real spaceflight conditions, we also assessed their effects in sμ*g* (comparison F vs. E). In this context, several neuronal activity‐related GO terms were enriched, including *postsynaptic specialization membrane, neuronal cell body,* and *distal axon*. In the presence of this stressor, we observed the upregulation of several antioxidant‐related genes that were also induced under normal gravitational load, such as *CLU* (log_2_ fold change 1.9), *FOXO1* (log_2_ fold change 2.0), and *GPNMB* (log_2_ fold change 3.0). In addition, with a log_2_ fold change of 3.7, we found the overexpression of *IGFBP6*, a gene involved in the response to insulin‐like growth factor.^[^ ^63^ ^]^ Its upregulation enhances IGF‐mediated trophic signaling, leading to the activation of pro‐survival pathways (e.g., Akt) and thereby increasing neuronal resilience.^[^ ^64^ ^]^ Another example is *PDGFRA*, which showed a log_2_ fold change of 1.9 and encodes for a receptor tyrosine kinase mediating platelet‐derived growth factor signaling.^[^ ^65^ ^]^ Its overexpression can strengthen trophic and protective responses against stressors such as oxidative stress, thereby promoting neuronal survival and modulating inflammation in the CNS, as previously observed in ischemic injury models.^[^ ^66^ ^]^ Finally, with a log_2_ fold change of 1.2, we noted the overexpression of *KCNMA1*, which encodes for a calcium‐activated potassium channel crucial for neuronal excitability.^[^ ^67^ ^]^ These channels are highly sensitive to oxidative stress, and their enhanced expression has been associated with improved regulation of neuronal excitability, reduced calcium overload, and protection against mitochondrial dysfunction in ischemia/reperfusion models.^[^ ^68^ ^]^

The analysis of cells exposed to the space environment and treated with PDNPs demonstrated the antioxidant potential of these nanostructures (comparison B vs. A). Further enrichment was also observed in terms such as *growth cone* and *neuron spine,* supporting the involvement of PDNPs in the modulation of pathways relevant to neuronal structure and function. Many genes that were previously found to be downregulated due to spaceflight stressors no longer showed reduced expression following PDNP treatment. For instance, genes associated with neuronal function, such as *RELN* and *SLC17A7* as well as those related to mitochondrial activity, including *MCU* and *NDUFAF5*, or dopaminergic metabolism, such as *SLC18A1* and *GNAL*, recovered their expression levels. Moreover, several other genes were found to be no longer downregulated following treatment with PDNPs. A particularly noteworthy example is the neuronal gene *LRRTM3*, which encodes for a transmembrane protein regulating presynaptic assembly at glutamatergic synapses.^[^ ^69^ ^]^ Decreased expression of *LRRTM3* was associated with decreased excitatory synapse density and impaired synaptic transmission, whereas PDNP treatment counteracted this downregulation.^[^ ^70^ ^]^ Also noteworthy is the *SHARPIN* gene, which encodes for a multifunctional protein essential for the activation of the NF‐κB signaling pathway.^[^ ^71^ ^]^ Diminished activation of this pathway in neurons can lead to increased susceptibility to oxidative stress and neuronal apoptosis, resulting in dysregulated neuroinflammation, a condition that may contribute to the progression of neurodegenerative conditions such as Alzheimer's and Parkinson's diseases.^[^ ^72^ ^]^ Similarly, several mitochondrial‐related genes regained ground‐like expression following PDNP administration. Among them, *SLC12A8*, encoding for a nicotinamide mononucleotide (NMN) transporter required for NAD^+^ biosynthesis, is of particular interest. Its downregulation was shown to impair NMN uptake, thereby lowering intracellular NAD^+^ levels and disrupting NAD^+^‐dependent mitochondrial metabolic pathways essential for ATP production.^[^ ^73^ ^]^ Another remarkable regulated marker is *PDE10A*, which encodes for phosphodiesterase 10A, an enzyme responsible for hydrolyzing the second messengers cAMP and cGMP in striatal neurons, thereby modulating dopaminergic signaling.^[^ ^74^ ^]^ Functional reduction of *PDE10A* was shown to alter dopamine metabolism in knockout mice, where an increased dopamine turnover in the striatum and a higher locomotor response to dopaminergic stimuli, (comparable to psychostimulant exposure) indicate dopaminergic transmission dysfunction.^[^ ^75^ ^]^

It is important to note, however, that not all genes downregulated by spaceflight stressors were fully restored by PDNP treatment. In several cases, downregulation was only partially mitigated. For example, previously observed genes such as *MBP*, *MICU3*, and *SHANK3* shifted from log_2_ fold changes of −5.3, −2.5, and −3.8, respectively, under spaceflight exposure, to −3.2, −2.0, and −3.1 following PDNP treatment.

Transcription of other genes also showed a partial recovery. Encoding for protocadherin 19, a neuronal adhesion molecule involved in synaptic network formation and intercellular signaling,^[^ ^76^ ^]^ *PCDH19* underwent a log_2_ fold change from −9.8 to −5.4. Decreased expression of this gene can compromise neuronal connectivity and synaptic transmission and has been associated with cognitive deficits.^[^ ^77^ ^]^ Similarly, *GABBR2*, encoding for a subunit of the GABA‐B receptor essential for maintaining the excitatory/inhibitory balance in the brain, underwent a log_2_ fold change from −10.1 to −5.0.^[^ ^78^ ^]^ Its downregulation was shown to diminish GABAergic signaling, leading to neuronal hyperexcitability and predisposition to neurological disorders.^[^ ^79^ ^]^ Finally, *NPAS3*, a transcription factor implicated in brain development, underwent a log_2_ fold change from −5.0 to −2.3. Notably, its reduced expression is known to impair both neurogenesis and neuronal metabolism.^[^ ^80^ ^]^

Among all the genes that underwent statistically significant downregulation due to exposure to the space environment, only 21% remained downregulated following treatment with PDNPs, and in many cases, the extent of downregulation was notably reduced. Overall, these findings seem to suggest a remarkable neuroprotective capacity of PDNPs, which not only counteracted the spaceflight‐induced downregulation of several key genes involved in neuronal signaling and mitochondrial metabolism, but also partially restored the expression of others, thereby preserving essential pathways for neuronal survival and function under extreme environmental stressors and even in the presence of microbial contaminants.

Beyond investigating the direct effects of PDNP treatment, we also assessed the nanoparticle rescue effect by comparing cells exposed to a specific stress and treated with PDNPs against control cells that experienced neither stress nor treatment. In the rescue condition mediated by the antioxidant nanoparticles, we observed the enrichment of fewer GO terms compared with the other comparisons. This reduction in enriched categories may indicate that the nanoparticle activity contributed to restoring cellular processes toward a state closer to the baseline physiological condition. In particular, when examining rescue under μ*g* (comparison B vs. C), we found that roughly 99% of the genes significantly downregulated by μ*g* alone were no longer downregulated in cultures supplemented with antioxidants. This indicates an almost complete rescue of neuronal damage by PDNPs in μ*g* conditions. In contrast, for CR exposure (comparison B vs. E), about 41% of the genes significantly downregulated by radiation alone regained ground expression levels in cultures supplemented with antioxidants. This partial rescue suggests that PDNPs mitigate, but do not fully reverse, radiation‐induced neuronal damage. We also evaluated gene upregulation driven by PDNP treatment. In the combined μ*g* and CR scenario (comparison B vs. G), several genes showed higher transcription, mirroring those upregulated in control cells treated with PDNPs and underscoring the nanoparticle beneficial effects toward neurons. Among these markers, with a log_2_ fold change of 7.7, we found *MT‐RNR2* that encodes for humanin, playing a role in neuronal protection from β‐amyloid and oxidative stress by inhibiting apoptosis and supporting mitochondrial function.^[^ ^81^ ^]^ We also found upregulation of *SGK1* (log_2_ fold change 1.5), a serine/threonine kinase, that regulates several targets (like ion channels, transporters, and transcription factors) to promote neuronal survival, enhance synaptic plasticity, and increase resistance to ischemic and oxidative insults.^[^ ^82^ ^]^ Finally, we found upregulation of *SLITRK6,* which shows a log_2_ fold change of 1.1 and encodes for a transmembrane protein that guides neurite outgrowth and synapse formation;^[^ ^83^ ^]^ typically, its upregulation boosts synapse number and maturation, improving neuronal connectivity and cognitive function in preclinical models.^[^ ^84^ ^]^

## 4 Conclusions

We showed that the combination of microgravity and cosmic radiation in space imposes severe oxidative stress on neuronal cells, leading to impaired synaptic function, mitochondrial energy deficits, and disrupted dopamine signaling. These molecular disturbances likely contribute to the motor and cognitive challenges faced by astronauts during long‐duration missions. Our data show that, thanks to their biocompatibility and strong antioxidant properties, PDNPs markedly reduce oxidative damage in neuronal cells and induce significant transcriptional changes both under spaceflight and ground conditions. In μ*g* alone, PDNPs rescue nearly all affected genes, and substantially mitigate radiation‐induced damage when both stressors (microgravity and cosmic radiation) are combined. Beyond spaceflight, PDNPs offer a rapid model for exploring treatments against neurodegenerative disorders driven by oxidative stress, such as Parkinson's disease. Future work will focus on validating efficacy in whole‐organism studies, testing in actual space missions, and initiating clinical trials on Earth. Integrating PDNPs with exercise, nutrition, and pharmacological strategies may unlock comprehensive countermeasures for maintaining brain health in extreme environments and aging populations.

## 5 Experimental Section

5.1

5.1.1

##### Nanoparticle Synthesis

PDNPs with a nominal diameter of 100 nm were synthesized by slightly modifying a previously published protocol from our group.^[^ ^85^ ^]^ Briefly, a mixture of 90 mL of ultrapure water, 40 mL of >96% ethanol, and 4 mL of ammonium hydroxide solution (30%, Sigma) was stirred gently at room temperature (RT) for 30 min. Subsequently, 10 mL of ultrapure water containing 0.5 g of dopamine hydrochloride (Sigma) was added to the solution; the resulting mixture was stirred continuously for 24 h. Then, the obtained nanoparticle dispersion was diluted 1:1 v/v with ethanol and centrifuged at 16 000 g for 30 min at 4 °C. The nanoparticle pellet was washed three times by centrifugation at the same speed and temperature, and then resuspended in sterile ultrapure water. PDNP concentration was determined gravimetrically by weighing freeze‐dried samples.

For cellular uptake experiments, PDNPs were fluorescently labeled with DiO (Vybrant Multicolor Cell‐Labeling Kit, Thermo Fisher Scientific) by incubating 5 mg of nanoparticles in 1 mL of ultrapure water with 10 μM of dye for 2 h under stirring. The labeled nanoparticles were washed three times with ultrapure water via centrifugation at 16 000 g to remove unbound dye. This labeling is based on non‐covalent adsorption of the lipophilic DiO dye onto the negatively charged, aromatic‐rich surface of polydopamine. This interaction is primarily driven by hydrophobic forces, electrostatic attraction between the cationic dye and the nanoparticle surface, and π–π stacking between aromatic domains of DiO and polydopamine.^[^ ^86^, ^87^ ^]^

##### Nanoparticle Characterization

The size and morphology of PDNPs were evaluated via SEM and TEM imaging, with subsequent image analysis by using Gwyddion software. For SEM imaging, 10 μL of a 100 μg mL^−1^ nanoparticle suspension was deposited onto a silicon substrate and air‐dried, followed by gold sputtering using a Quorum Tech Q150RES Gold Sputter Coater (30 mA, 60 s). SEM images were acquired using a Helios NanoLab 600i FIB/SEM Dual‐Beam system (FEI). For TEM, 10 μL of the nanoparticle suspension was applied to a 150‐mesh copper grid coated with carbon, and bright‐field images were collected by using a JEM‐1400Plus system (JEOL) equipped with a LaB_6_ thermionic source operating at 120 kV.

DLS measurements were carried out by using a Malvern Zetasizer Nano ZS90 to determine *D*_h_, PDI, and *ζ*‐potential. *D*_h_ and PDI were measured in dispersions (100 μg mL^−1^) by using polystyrene cuvettes, while *ζ*‐potential was evaluated in folded capillary cells.

A nanoparticle stability study was conducted by incubating PDNPs (100 μg mL^−1^) in differentiation medium composed of high‐glucose Dulbecco's modified Eagle's medium/F‐12 with 15 mM HEPES (DMEM/F12, Gibco), supplemented with 2% heat‐inactivated fetal bovine serum (FBS, Gibco), 1% L‐glutamine (200 mM stock, Gibco), 10 μM retinoic acid (RA, Thermo Scientific), and 1% penicillin‐streptomycin (100 IU/mL penicillin, 100 μg mL^−1^ streptomycin, Gibco). *D*_h_ and PDI values were monitored over 1 h (with measurements every 10 min) and over 10 days (with measurements every 48 h).

The antioxidant activity of PDNPs was assessed by using the Total Antioxidant Capacity Assay Kit (Sigma). Briefly, 100 μL of a 2 μg mL^−1^ PDNP dispersion in ultrapure water were mixed with 100 μL of Cu^2+^ working solution and incubated in the dark at RT for 90 min. Absorbance was read at 570 nm by using a Victor X3 Multilabel Plate Reader (Perkin Elmer). Trolox‐equivalent antioxidant activity was determined via a calibration curve. For comparative purposes, the same protocol was applied to standard antioxidant compounds, namely 100 μM of ascorbic acid, 10 μM of tannic acid, and 100 μM of idebenone, to benchmark the intrinsic antioxidant capacity of PDNPs under matched conditions.

##### Cell Culture

SH‐SY5Y neuroblastoma cells (HTL95013, ICLC) were maintained under proliferative conditions in DMEM/F‐12, supplemented with 15 mM HEPES, 10% heat‐inactivated FBS, 1% L‐glutamine, and 1% penicillin‐streptomycin. For neuronal differentiation, the proliferation medium was replaced with differentiation medium.

##### Nanoparticle/Cell Interaction

In vitro assays for investigating nanoparticle‐cell interactions were conducted by using differentiated SH‐SY5Y cells cultured under either 1*g* or sμ*g* conditions. sμ*g* was achieved by using an RPM (Airbus 2.0), operated at a frame speed of 25–60 s^−1^. The home position of the RPM was defined as the configuration in which the sample holder was aligned with the 1*g* vector, with both frames positioned perpendicular to this vector.

The effect of PDNPs on cell viability was assessed by using the Quant‐iT PicoGreen dsDNA Assay Kit (Invitrogen). Neuron‐like cells were seeded in 96‐well plates (Corning) at a density of 10 000 cells cm^−2^ and incubated in proliferation medium for 24 h. Cells were then exposed to increasing concentrations of PDNPs (0–200 μg mL^−1^) in differentiation medium for 48 or 96 h. After treatment, cells were washed with Dulbecco's phosphate‐buffered saline (DPBS, Sigma) and underwent three freeze–thaw cycles (−80 °C to 37 °C) in 100 μL ultrapure water for lysis. The PicoGreen assay was performed by mixing the lysate with PicoGreen reagent and Tris‐ethylenediaminetetraacetic acid (EDTA) buffer in black 96‐well polystyrene plates (Corning Costar), following the manufacturer's instructions. Fluorescence was measured (*λ*_ex_ 485 nm, *λ*_em_ 535 nm) by using a Victor X3 Multilabel Plate Reader (Perkin Elmer).

To visualize nanoparticle uptake, cells were seeded in CELLview dishes (VWR) at 10 000 cells cm^−2^ and incubated overnight. SH‐SY5Y were then treated with 20 μg mL^−1^ DiO‐labeled PDNPs for either 6, 48, or 96 h in differentiation medium. After treatment, cells were fixed with 4% paraformaldehyde (PFA) in DPBS at 4 °C for 20 min, then permeabilized with 0.1% Triton in DPBS, saturated with 10% goat serum in DPBS, and stained with 5 μg mL^−1^ Hoechst and 2.5 μg mL^−1^ TRITC‐phalloidin in DPBS at 37 °C for 1 h. Imaging was performed by using a Nikon C2s confocal microscope with a 60 × oil‐immersion objective.

Quantitative analysis of PDNPs uptake was carried out via flow cytometry. Neuron‐like cells were seeded in 24‐well plates at a density of 10 000 cells cm^−2^ and incubated overnight. After exposure to 20 μg mL^−1^ DiO‐PDNPs in differentiation medium for 6, 48, or 96 h, cells were washed with DPBS, trypsinized, and analyzed on a CytoFLEX cytometer (*λ*_ex_ 488 nm, *λ*_em_ 525 ± 40 nm; Beckman Coulter).

To evaluate the antioxidant effect of PDNPs in neuron‐like cells, we used the GSH Assay Kit (Abcam). Cells were seeded in 24‐well plates at a density of 10 000 cells cm^−2^ and incubated for 24 h in proliferation medium. Cultures were then treated for 96 h with 20 μg mL^−1^ PDNPs, 30 nM TBH as a pro‐oxidant agent, or both, resulting into four experimental groups: untreated control, PDNP‐added, TBH‐added, and PDNPs + TBH‐added. At the end of treatment, cells were harvested and processed according to the manufacturer's protocol. Absorbance was measured at 415 nm using a Victor X3 Multilabel Plate Reader (Perkin Elmer).

PDNP antioxidant effect was also assessed by evaluating the intracellular oxidative stress levels. Neuron‐like cells were seeded in 24‐well plates at a density of 10 000 cells cm^−2^ and incubated for 24 h in proliferation medium, and then treated for 96 h with 20 μg mL^−1^ of PDNPs. After treatment, cells were washed with DPBS and stained with 2.5 μM CellROX Green reagent (ThermoFisher) for 30 min at 37 °C in phenol red‐free medium. Following staining, cells were detached by using trypsin, resuspended in phenol red‐free medium, and divided into different experimental groups (Control, PDNPs, TBH, and PDNPs + TBH). TBH was added for oxidative stress induction to designated samples at a final concentration of 5.0 mM, and the cell suspensions were incubated for 30 min at 37 °C. Fluorescence was immediately analyzed by flow cytometry (CytoFLEX, Beckman Coulter; *λ*_ex_ 488 nm, *λ*_em_ 525 ± 40 nm) to assess ROS levels.

Eventually, PDNP antioxidant effect was also assessed in mitochondria by evaluating mitochondrial ROS levels and mitochondrial membrane potential levels. Neuron‐like cells were seeded and treated as previously described, then superoxide levels were assessed using MitoSOX Red reagent (ThermoFisher), while variations in mitochondrial membrane potential were assessed by using tetramethyl rhodamine methyl ester (Life Technologies). After PDNP and TBH treatments, cells were incubated with 2.5 μM MitoSOX Red for 10 min at 37 °C, or 1 μM rhodamine for 30 min at 37 °C. Fluorescence was immediately analyzed by flow cytometry (CytoFLEX, Beckman Coulter; *λ*_ex_ 488 nm, *λ*_em_ 525 ± 40 nm) to assess ROS levels and effects on mitochondrial membrane potential.

To assess the influence of PDNPs on neuronal differentiation, neuron‐like cells were seeded at low density (1 000 cells cm^−2^) in CELLview dishes. After 24 h in proliferation medium, cells were cultured for 96 h in differentiation medium, with or without 20 μg mL^−1^ PDNPs. Cells were then fixed in 4% PFA in DPBS at 4 °C for 20 min and stained for tubulin β‐III. The staining protocol included permeabilization with 0.1% Triton in DPBS, saturation with 10% goat serum in DPBS, and 3 h of incubation in 10% goat serum with 0.3 μg mL^−1^ rabbit antitubulin β‐III antibody (Sigma), followed by 40 min of incubation in 10% goat serum with 10 μg mL^−1^ goat anti‐rabbit Alexa Fluor 488‐conjugated secondary antibody and 5 μg mL^−1^ Hoechst 33342. After three DPBS rinses, cells were imaged with a Nikon Eclipse Ti epifluorescence microscope (10× objective), and image analysis was performed with ImageJ.

##### In‐Flight Experiment

Neuron‐like cells were seeded at a density of 9,000 cells cm^−2^ onto Thermanox coverslips (10.5 × 22.0 mm, Nunc) placed in ad hoc prepared silicone multiwell plates at ≈L‐120 h. A schematic overview of the experimental timeline is provided in Figure S11a, Supporting Information. At L‐72 h, the samples were transferred into 12 EUs (KEU‐ST, developed by Kayser Italia and flight‐qualified for use on board the ISS, providing the first level of required containment). Each EU contained five reservoirs, filled as follows with a volume of 1.3 mL: reservoirs 1 and 2 with a HEPES‐buffered differentiation medium, either supplemented or not with PDNPs at a final concentration of 20 μg mL^−1^; reservoirs 3 and 4 with DPBS for cell rinsing before fixation; reservoir 5 with RNAlater (Ambion AM7020) for fixation and nucleic acid preservation. A schematic representation of the EU fluidic system is shown in Figure S11b, Supporting Information. Each EU (*n* = 3 per experimental group) was enclosed within an EC (KIC‐SL, developed by Kayser Italia and qualified for spaceflight), which provided the second level of required containment, and interfaced with the ESA Kubik incubator on board the ISS. Selected ECs were also equipped with iButton data loggers for temperature monitoring. From handover to hardware integration into Kubik, samples were kept under passive thermal conditioning within +25 to +30 °C. The experimental payload was uploaded via Cygnus/Antares vehicle (NG‐18 increment). Within 24 h from Cygnus docking to the ISS, the ECs were positioned into the Kubik incubator (previously set at +37 °C) as shown in Figure S12, Supporting Information, and the first of the five fluidic activations was automatically executed. Release of reservoirs 1 and 2 content (differentiation medium with or without PDNPs) occurred at L + 73 h and L + 121 h, respectively. Release of reservoirs 3 and 4 content (DPBS) was instead conducted at L + 169 h, with a 2‐min interval. Release of reservoir 5 content (fixative) occurred 2 min later. At 2 h from fixation, ECs were transferred to the −80 °C Laboratory Freezer for ISS (MELFI), and cold‐stowed at −80 °C until return to Earth. The whole experiment was then replicated on Earth by using an RPM to simulate μ*g* conditions.

##### Transcriptomic Analyses

The investigation of PDNP effects under μ*g* and CR exposures resulted in eight experimental groups: A (‐PDNPs, μ*g*, +CR); B (+PDNPs, μ*g*, +CR); C (‐PDNPs, s1*g*, +CR); D (+PDNPs, s1*g*, +CR); E (‐PDNPs, sμ*g*, ‐CR); F (+PDNPs, sμ*g*, ‐CR); G (‐PDNPs, 1*g*, ‐CR); H (+PDNPs, 1*g*, ‐CR). Of all possible pairwise comparisons, the nine following were selected (the control group is listed as second in each pair): H vs. G was considered to evaluate the intrinsic effect of PDNPs (PDNPs‐only condition); B vs. A to assess the neuroprotective effects of PDNPs under spaceflight conditions; B vs. G to assess the rescue performed by PDNPs under spaceflight conditions; A vs. G to evaluate spaceflight stressors; A vs. C to distinguish the contribution of μ*g* alone; C vs. G to distinguish the contribution of CR alone; F vs. E to evaluate the impact of PDNPs in the presence of μ*g* (simulated); B vs. C to assess the rescue performed by PDNPs against μ*g*; B vs. E to assess the rescue performed by PDNPs against CR.

Following de‐integration of hardware resulting from the space experiment and from the ground‐based control experiment, cell cultures were imaged by using phase‐contrast optical microscopy (Nikon Eclipse Ti), and temperature profiles were retrieved from data loggers to verify maintenance of conditions compatible with cell viability. Cells were then transferred to 2 mL tubes, covered with their supernatants (comprising over 70% RNAlater), and centrifuged at 16 000 g for 15 min at 4 °C to isolate cell pellets. RNA extraction and purification were performed by using the MirVana PARIS Kit (Ambion AM1556), based on the manufacturer's instructions. RNA integrity was investigated by agarose gel electrophoresis, that was conducted with 100 ng of RNA (quantified by using a NanoDrop 2000 spectrophotometer, Thermo Scientific) on 0.8% agarose (Sigma‐Aldrich) in 1 × Tris‐Borate‐EDTA (TBE) gel, added with 5 μl of ethidium bromide, under 0.5 × TBE as running buffer at 120 V for 45 min in an electrophoretic cell (Bio‐Rad). Gel imaging was performed with Chemidoc XRS + transilluminator (Bio‐Rad).

RNA samples were then processed by using the GeneWiz Ultra‐Low Input RNA‐Seq workflow (https://web.genewiz.com/ultra‐low‐input‐case‐study). Libraries were prepared with the NEBNext Ultra RNA II Directional RNA Library Prep Kit for Illumina (New England Biolabs, #E7760/E7765). This included poly(A) enrichment with NEBNext Poly(A) mRNA Magnetic Isolation Module (NEB #7490), fragmentation, and reverse transcription into cDNA (first and second strands), followed by end repair, 5′ phosphorylation, polyadenylation, adapter ligation, and PCR amplification. Final library yield was quantified by using the Qubit DNA assay (Thermo Fisher Scientific) and NanoDrop, while fragment size distribution was evaluated with the 2100 Bioanalyzer (Agilent). Real‐time PCR was used to quantify viable sequencing templates. Sequencing was performed by using an Illumina Illumina NovaSeq 6000 system in paired‐end configuration. Raw reads were converted to FASTQ files.

##### Bioinformatic Processing

FASTQ file quality was assessed with FastQC. Cleaned reads from standard libraries were aligned to the human reference genome (hg38, Gencode)^[^ ^88^ ^]^ by using STAR aligner v2.7.11b,^[^ ^89^ ^]^ and corresponding BAM files were generated. Gene‐level counts were computed by using featureCounts^[^ ^90^ ^]^ from the Subread package v2.18.0,^[^ ^91^ ^]^ considering only unique reads within exonic regions. Differential gene expression analysis was carried out by using DESeq2 v1.44.0.^[^ ^92^ ^]^ Transcripts were considered differentially expressed if they met the following criteria: absolute log_2_ fold change (FC) > abs (1) and adjusted *p*‐value (from Wald test) < 0.05. This analysis yielded 9 DEGs lists, alongside heatmaps and volcano plots generated by using pheatmap v1.0.12 and EnhancedVolcano v1.22.0, respectively.^[^ ^93^ ^]^ To interpret the biological impact of the different experimental conditions, GO enrichment analysis was performed by using clusterProfiler v4.12.0,^[^ ^94^ ^]^ applying a significance threshold of 0.05 for both *p*‐ and *q*‐values.

##### Statistical Analysis

All statistical analyses were carried out using *R* software. Data normality was assessed by using the Shapiro‐Wilk test. For normally distributed datasets, comparisons were made by using ANOVA followed by LSD posthoc testing with Bonferroni correction, and results were reported as mean ± standard deviation. Non‐normally distributed data were analyzed by using the Kruskal–Wallis test followed by Wilcoxon posthoc testing with Holm correction, and results were expressed as median ± 95% confidence interval. All experiments were performed in triplicate (*n* = 3), unless otherwise indicated. For transcriptomic statistical analyses, please refer to the specific experimental section.

## Supporting Information

PDNP stability evaluation; PDNP uptake in 1*g* conditions; PDNP uptake in sμ*g* conditions; GSH level analysis; cytosolic ROS level evaluation; mitochondrial ROS level evaluation; mitochondrial membrane potential assessment; thermal profile of the in‐flight experiment; RNA agarose gel electrophoresis; GO enrichment analysis; timeline and EU setup; EU assembly and placement in the incubator; comparative antioxidant performance of standard compounds; and DEG summary.

## Conflict of Interest

The authors declare no conflict of interest.

## Supporting information

Supplementary Material

## Data Availability

Omics data were deposited in NCBI Gene Expression Omnibus database and can be accessed through the GEO Series accession number GSE290701. All other data are available upon request to the authors.

## References

[1] C. A. Montesinos , R. Khalid , O. Cristea , J. S. Greenberger , M. W. Epperly , J. A. Lemon , D. R. Boreham , D. Popov , G. Gorthi , N. Ramkumar , J. A. Jones , Life 2021, 11, 829. 34440577 10.3390/life11080829 PMC8398261

[2] U. Gupta , S. Baig , A. Majid , S. M. Bell , Life Sci. Space Res. 2023, 36, 105. 10.1016/j.lssr.2022.09.003 36682819

[3] E. Giedzinski , R. Rola , J. R. Fike , C. L. Limoli , Radiat. Res. 2005, 164, 540. 16187784 10.1667/rr3369.1

[4] S. Manoharan , G. J. Guillemin , R. S. Abiramasundari , M. M. Essa , M. Akbar , M. D. Akbar , Oxid. Med. Cell Longev. 2016, 2016, 8590578. 28116038 10.1155/2016/8590578 PMC5223034

[5] F. L. Wuyts , C. Deblieck , C. Vandevoorde , M. Durante , Nat. Rev. Neurosci. 2025, 26, 354. 40247135 10.1038/s41583-025-00923-4

[6] V. Mann , A. Sundaresan , M. F. J. Doursout , S. Devakottai , Spaceflight and the Central Nervous System: Clinical and Scientific Aspects, Springer International, Cham 2022, p. 23.

[7] K. MANDA , M. Ueno , K. Anzai , Behav. Brain Res. 2008, 187, 387. 18006086 10.1016/j.bbr.2007.09.033

[8] G. Sprugnoli , Y. D. Cagle , E. Santarnecchi , JAMA Neurol. 2020, 77, 157. 31764952 10.1001/jamaneurol.2019.4003 PMC8976449

[9] V. K. Parihar , B. D. Allen , K. K. Tran , N. N. Chmielewski , B. M. Craver , V. Martirosian , J. M. Morganti , S. Rosi , R. Vlkolinsky , M. M. Acharya , G. A. Nelson , A. R. Allen , C. L. Limoli , Antioxid. Redox Signal. 2015, 22, 78. 24949841 10.1089/ars.2014.5929 PMC4270160

[10] O. V. Belov , K. V. Belokopytova , V. S. Kudrin , A. G. Molokanov , A. S. Shtemberg , A. S. Bazyan , Physica Med. 2019, 57, 7. 10.1016/j.ejmp.2018.12.003 30738534

[11] N. Ali , A. Beheshti , G. Hampikian , NPJ Microgravity 2025, 11, 1. 39753605 10.1038/s41526-024-00457-6 PMC11698718

[12] N. Ali , A. Beheshti , G. Hampikian , Unveiling Parkinson's Disease‐like Changes Triggered by Spaceflight 2024.

[13] I. Tyuryaeva , O. Lyublinskaya , Int. J. Mol. Sci. 2023, 24, 9303. 37298254 10.3390/ijms24119303 PMC10252755

[14] M. Battaglini , M. Emanet , A. Carmignani , G. Ciofani , Nano Today 2024, 55, 102151.

[15] T. T. Zhu , H. Wang , H. W. Gu , L.‐S. Ju , X.‐M. Wu , W.‐T. Pan , M.‐M. Zhao , J.‐J. Yang , P.‐M. Liu , J. Nanobiotechnol. 2023, 21, 1. 10.1186/s12951-023-01807-4 PMC9913011 36765377

[16] M. Battaglini , A. Marino , A. Carmignani , C. Tapeinos , V. Cauda , A. Ancona , N. Garino , V. Vighetto , G. La Rosa , E. Sinibaldi , G. Ciofani , ACS Appl. Mater. Interfaces 2020, 12, 35782. 32693584 10.1021/acsami.0c05497 PMC8009471

[17] M. Battaglini , A. Carmignani , C. Martinelli , J. Colica , A. Marino , S. Doccini , V. Mollo , F. Santoro , M. Bartolucci , A. Petretto , F. M. Santorelli , G. Ciofani , Biomater. Sci. 2022, 10, 3770. 35635043 10.1039/d2bm00729k

[18] C. Jian , Y. Hong , H. Liu , Q. Yang , S. Zhao , Int. J. Pharm. 2025, 669, 125087. 39675536 10.1016/j.ijpharm.2024.125087

[19] G. Pani , M. Verslegers , R. Quintens , N. Samari , L. de Saint‐Georges , P. van Oostveldt , S. Baatout , M. A. Benotmane , PLoS One 2016, 11, e0155260. 27203085 10.1371/journal.pone.0155260 PMC4874625

[20] E. A. Kulikova , V. A. Kulikov , N. A. Sinyakova , A. V. Kulikov , N. K. Popova , Neurosci. Lett. 2017, 640, 88. 28088578 10.1016/j.neulet.2017.01.023

[21] N. K. Popova , A. V. Kulikov , E. M. Kondaurova , A. S. Tsybko , E. A. Kulikova , I. B. Krasnov , B. S. Shenkman , E. Y. Bazhenova , N. A. Sinyakova , V. S. Naumenko , Mol. Neurobiol. 2015, 51, 1443. 25084757 10.1007/s12035-014-8821-7

[22] A. Carmignani , M. Battaglini , M. Bartolucci , A. Petretto , M. Prato , G. Ciofani , Mater. Des. 2024, 239, 112825.

[23] G. G. Genchi , A. Degl'innocenti , C. Martinelli , M. Battaglini , D. De Pasquale , M. Prato , S. Marras , G. Pugliese , F. Drago , A. Mariani , M. Balsamo , V. Zolesi , G. Ciofani , ACS Appl. Mater. Interfaces 2021, 13, 40200. 34410709 10.1021/acsami.1c14176 PMC8414486

[24] T. P. Stein , Nutrition 2002, 18, 867. 12361781 10.1016/s0899-9007(02)00938-3

[25] G. G. Genchi , V. Mollo , M. Battaglini , M. Belenli Gümüş , A. Marino , M. Prato , S. Marras , F. Drago , G. Pugliese , F. Santoro , G. Ciofani , ACS Appl. Nano Mater. 2023, 6, 10853.

[26] A. D. Filev , E. S. Ershova , E. A. Savinova , A. M. Kalakov , N. N. Veiko , P. E. Umriukhin , S. V. Kostyuk , Int. J. Biol. Biomed. Eng. 2021, 15, 371.

[27] A. Carmignani , T. Yamazaki , M. Battaglini , C. Q. Vu , A. Marino , S. Takayanagi‐Kiya , T. Kiya , A. Armirotti , A. Di Fonzo , S. Arai , G. Ciofani , ACS Nano 2025, 19, 16267. 40270300 10.1021/acsnano.5c04181 PMC12060647

[28] H. Zhao , Z. Zeng , L. Liu , J. Chen , H. Zhou , L. Huang , J. Huang , H. Xu , Y. Xu , Z. Chen , Y. Wu , W. Guo , J. H. Wang , J. Wang , Z. Liu , Nanoscale 2018, 10, 6981. 29610822 10.1039/c8nr00838h

[29] S. J. Park , S. Onizuka , M. Seki , Y. Suzuki , T. Iwata , K. Nakai , BMC Biol. 2019, 17, 72. 31519179 10.1186/s12915-019-0690-0 PMC6743104

[30] J. Gao , S. Wu , J. Yang , T. Ye , J. Yang , W. Shen , X. Chen , L. Huang , R. Pang , P. Lin , J. Lin , Y. Zhou , W. Wang , T. Tan , Transl. Psychiatry 2025, 15, 1. 40783396 10.1038/s41398-025-03505-1 PMC12335487

[31] A. Kathuria , P. Nowosiad , R. Jagasia , S. Aigner , R. D. Taylor , L. C. Andreae , N. J. F. Gatford , W. Lucchesi , D. P. Srivastava , J. Price , Mol. Psychiatry 2018 23:3 2017, 23, 735. 28948968 10.1038/mp.2017.185 PMC5822449

[32] X. Du , J. Li , M. Li , X. Yang , Z. Qi , B. Xu , W. Liu , Z. Xu , Y. Deng , Cell Biosci 2020, 10, 1. 32158532 10.1186/s13578-020-00393-4 PMC7057577

[33] S. M. Wojcik , J. S. Rhee , E. Herzog , A. Sigler , R. Jahn , S. Takamori , N. Brose , C. Rosenmund , Proc. Natl. Acad. Sci. U S A 2004, 101, 7158. 15103023 10.1073/pnas.0401764101 PMC406482

[34] D. D’Angelo , R. Rizzuto , Biomolecules 2023, 13, 1304. 37759703 10.3390/biom13091304 PMC10526485

[35] L. Bierhansl , L. Gola , V. Narayanan , A. Dik , S. G. Meuth , H. Wiendl , S. Kovac , Mol. Neurobiol. 2024, 61, 9529. 38652352 10.1007/s12035-024-04148-x PMC11496325

[36] C. Sugiana , D. J. Pagliarini , M. McKenzie , D. M. Kirby , R. Salemi , K. K. Abu‐Amero , H. H. M. Dahl , W. M. Hutchison , K. A. Vascotto , S. M. Smith , R. F. Newbold , J. Christodoulou , S. Calvo , V. K. Mootha , M. T. Ryan , D. R. Thorburn , Am. J. Hum. Genet. 2008, 83, 468. 18940309 10.1016/j.ajhg.2008.09.009 PMC2561934

[37] Y. Wen , G. Lu , L. Qiao , Y. Li , Mol. Genet. Genomic Med. 2021, 10, e1852. 34964562 10.1002/mgg3.1852 PMC8801144

[38] H. O. Lawal , D. E. Krantz , Mol. Aspects Med. 2013, 34, 360. 23506877 10.1016/j.mam.2012.07.005 PMC3727660

[39] F. W. Lohoff , G. V. Carr , B. Brookshire , T. N. Ferraro , I. Lucki , Brain Res. 2019, 1712, 151. 30685272 10.1016/j.brainres.2019.01.029

[40] N. Di Donato , R. Guerrini , C. J. Billington , A. J. Barkovich , P. Dinkel , E. Freri , M. Heide , E. S. Gershon , T. S. Gertler , R. J. Hopkin , S. Jacob , S. K. Keedy , D. Kooshavar , P. J. Lockhart , D. R. Lohmann , I. G. Mahmoud , E. Parrini , E. Schrock , G. Severi , A. E. Timms , R. I. Webster , M. J. H. Willis , M. S. Zaki , J. G. Gleeson , R. J. Leventer , W. B. Dobyns , Brain 2022, 145, 3274. 35769015 10.1093/brain/awac164 PMC9989350

[41] A. Guidotti , D. R. Grayson , H. J. Caruncho , Front Cell. Neurosci. 2016, 10, 89. 27092053 10.3389/fncel.2016.00089 PMC4820443

[42] P. Feng , A. A. Akladious , Y. Hu , Psychiatry Res. 2016, 243, 210. 27423632 10.1016/j.psychres.2016.06.052

[43] A. Katzman , C. M. Alberini , Curr. Opin. Neurobiol. 2017, 48, 122. 29278843 10.1016/j.conb.2017.12.003 PMC5825275

[44] F. Palmieri , Pflugers Arch. 2004, 447, 689. 14598172 10.1007/s00424-003-1099-7

[45] G. C. Shaw , J. J. Cope , L. Li , K. Corson , C. Hersey , G. E. Ackermann , B. Gwynn , A. J. Lambert , R. A. Wingert , D. Traver , N. S. Trede , B. A. Barut , Y. Zhou , E. Minet , A. Donovan , A. Brownlie , R. Balzan , M. J. Weiss , L. L. Peters , J. Kaplan , L. I. Zon , B. H. Paw , Nature 2006, 440, 96. 16511496 10.1038/nature04512

[46] S. Longueville , R. Yuan , C. Naon , E. Valjent , A. Pelosi , E. Roze , L.‐L. Mariani , J.‐A. Girault , D. Herve , Characterization of Mice with Cell Type‐Specific Gnal Loss of Function Provides Insights on GNAL‐Linked Dystonia. bioRxiv 2025, 2025.07.02.662743. 10.1016/j.nbd.2025.107071 40902679

[47] T. Fuchs , R. Saunders‐Pullman , I. Masuho , M. S. Luciano , D. Raymond , S. Factor , A. E. Lang , T. W. Liang , R. M. Trosch , S. White , E. Ainehsazan , D. Hervé , N. Sharma , M. E. Ehrlich , K. A. Martemyanov , S. B. Bressman , L. J. Ozelius , Nat. Genet. 2013, 45, 88. 23222958 10.1038/ng.2496 PMC3530620

[48] C. Rohr , J. Prestel , L. Heidet , H. Hosser , W. Kriz , R. L. Johnson , C. Antignac , R. Witzgall , J. Clin. Invest. 2002, 109, 1073. 11956245 10.1172/JCI13961 PMC150943

[49] L. Zhang , J. Xiong , H. M. Li , X. Li , X. Yu , Y. Liang , H. Sun , S. D. Yang , M. Shao , Pathol. Res. Pract. 2025, 269, 155936. 40174274 10.1016/j.prp.2025.155936

[50] O. Nicole , E. Pacary , Int. J. Mol. Sci. 2020, 21, 7272. 33019657 10.3390/ijms21197272 PMC7582470

[51] E. A. Albadawi , Neurosci. J. 2025, 30, 5. 10.17712/nsj.2025.1.20240052 PMC11753596 39800422

[52] G. J. Duncan , T. J. Simkins , B. Emery , Front. Cell Dev. Biol. 2021, 9, 653101. 33763430 10.3389/fcell.2021.653101 PMC7982542

[53] V. Tran , N. Carpo , S. Shaka , J. Zamudio , S. Choi , C. Cepeda , A. Espinosa‐Jeffrey , Life 2022, 12, 797. 35743828 10.3390/life12060797 PMC9224676

[54] M. Patron , V. Granatiero , J. Espino , R. Rizzuto , D. De Stefani , Cell Death Differ. 2018, 26, 179. 29725115 10.1038/s41418-018-0113-8 PMC6124646

[55] G. Bastioli , S. Piccirillo , L. Graciotti , M. Carone , G. Sprega , O. Taoussi , A. Preziuso , P. Castaldo , Cells 2024, 13, 1301. 39120330 10.3390/cells13151301 PMC11311461

[56] S. S. Stojilkovic , S. J. Sokanovic , S. Constantin , Front. Endocrinol. Lausanne 2025, 16, 1531723. 39926347 10.3389/fendo.2025.1531723 PMC11802530

[57] S. C. Daubner , T. Le , S. Wang , Arch. Biochem. Biophys. 2010, 508, 1. 21176768 10.1016/j.abb.2010.12.017 PMC3065393

[58] X. Huang , H. Chen , Y. Xie , Z. Cao , X. Lin , Y. Wang , Stem Cells Int. 2019, 2019, 2120453. 31781234 10.1155/2019/2120453 PMC6875375

[59] J. Wang , H. Fröhlich , F. B. Torres , R. L. Silva , G. Poschet , A. Agarwal , G. A. Rappold , Proc Natl Acad Sci U S A 2022, 119, e2112852119. 35165191 10.1073/pnas.2112852119 PMC8872729

[60] E. M. Foster , A. Dangla‐Valls , S. Lovestone , E. M. Ribe , N. J. Buckley , Front Neurosci 2019, 13, 164. 30872998 10.3389/fnins.2019.00164 PMC6403191

[61] J. M. Gregory , D. R. Whiten , R. A. Brown , T. P. Barros , J. R. Kumita , J. J. Yerbury , S. Satapathy , K. McDade , C. Smith , L. M. Luheshi , C. M. Dobson , M. R. Wilson , Acta Neuropathol. Commun. 2017, 5, 81. 29115989 10.1186/s40478-017-0481-1 PMC5678579

[62] X. Li , J. Xu , W. Su , L. Su , X. Chen , J. Yang , X. Lin , L. Yang , Cell Transplant 2024, 33, 09636897241233040. 38400732 10.1177/09636897241233040 PMC10894544

[63] A. R. D. Coda , A. Liso , F. Bellanti , Biology 2025, 14, 747. 40723309 10.3390/biology14070747 PMC12292253

[64] H. J. Jeon , J. Park , J. H. Shin , M. S. Chang , Int. J. Mol. Med. 2017, 40, 1860. 29039467 10.3892/ijmm.2017.3173 PMC5716453

[65] S. Sil , P. Periyasamy , A. Thangaraj , E. T. Chivero , S. Buch , Mol. Aspects Med. 2018, 62, 63. 29409855 10.1016/j.mam.2018.01.006 PMC6003857

[66] Z. Tang , P. Arjunan , C. Lee , Y. Li , A. Kumar , X. Hou , B. Wang , P. Wardega , F. Zhang , L. Dong , Y. Zhang , S. Z. Zhang , H. Ding , R. N. Fariss , K. G. Becker , J. Lennartsson , N. Nagai , Y. Cao , X. Li , J. Exp. Med. 2010, 207, 867. 20231377 10.1084/jem.20091704 PMC2856029

[67] A. Hermann , G. F. Sitdikova , T. M. Weiger , Biomolecules 2015, 5, 1870. 26287261 10.3390/biom5031870 PMC4598779

[68] E. Soltysinska , B. H. Bentzen , M. Barthmes , H. Hattel , A. B. Thrush , M. E. Harper , K. Qvortrup , F. J. Larsen , T. A. Schiffer , J. Losa‐Reyna , J. Straubinger , A. Kniess , M. B. Thomsen , A. Brüggemann , S. Fenske , M. Biel , P. Ruth , C. Wahl‐Schott , R.C. Boushel , S. P. Olesen , R. Lukowski , PLoS One 2014, 9, e103402. 25072914 10.1371/journal.pone.0103402 PMC4114839

[69] J. Kim , D. Park , N. Y. Seo , T. H. Yoon , G. H. Kim , S. H. Lee , J. Seo , J. W. Um , K. J. Lee , J. Ko , Proc. Natl. Acad. Sci. U S A 2022, 119, e2110196119. 35022233 10.1073/pnas.2110196119 PMC8784129

[70] J. W. Um , T. Y. Choi , H. Kang , Y. S. Cho , G. Choii , P. Uvarov , D. Park , D. Jeong , S. Jeon , D. Lee , H. Kim , S. H. Lee , Y. C. Bae , S. Y. Choi , M. S. Airaksinen , J. Ko , Cell Rep. 2016, 14, 808. 26776509 10.1016/j.celrep.2015.12.081

[71] C. Sala , V. Piëch , N. R. Wilson , M. Passafaro , G. Liu , M. Sheng , Neuron 2001, 31, 115. 11498055 10.1016/s0896-6273(01)00339-7

[72] B. Kaltschmidt , L. P. Helweg , J. F. W. Greiner , C. Kaltschmidt , Front. Mol. Neurosci. 2022, 15, 954541. 35983068 10.3389/fnmol.2022.954541 PMC9380593

[73] A. Grozio , K. F. Mills , J. Yoshino , S. Bruzzone , G. Sociali , K. Tokizane , H. C. Lei , R. Cunningham , Y. Sasaki , M. E. Migaud , S. I. Imai , Nat. Metab. 2019, 1, 47. 31131364 10.1038/s42255-018-0009-4 PMC6530925

[74] C. M. Macmullen , K. Vick , R. Pacifico , M. Fallahi‐Sichani , R. L. Davis , Transl. Psychiatry 2016, 6, e742. 26905414 10.1038/tp.2016.3 PMC4872433

[75] J. A. Siuciak , S. A. McCarthy , D. S. Chapin , A. N. Martin , J. F. Harms , C. J. Schmidt , Neuropharmacology 2008, 54, 417. 18061215 10.1016/j.neuropharm.2007.10.009

[76] J. J. T. Van Harssel , S. Weckhuysen , M. J. A. Van Kempen , K. Hardies , N. E. Verbeek , C. G. F. De Kovel , W. B. Gunning , E. Van Daalen , M. V. De Jonge , A. C. Jansen , R. J. Vermeulen , W. F. M. Arts , H. Verhelst , A. Fogarasi , J. F. De Rijk‐Van Andel , A. Kelemen , D. Lindhout , P. De Jonghe , B. P. C. Koeleman , A. Suls , E. H. Brilstra , Neurogenetics 2013, 14, 23. 23334464 10.1007/s10048-013-0353-1

[77] D. T. Pederick , C. C. Homan , E. J. Jaehne , S. G. Piltz , B. P. Haines , B. T. Baune , L. A. Jolly , J. N. Hughes , J. Gecz , P. Q. Thomas , Sci. Rep. 2016, 6, 26765. 27240640 10.1038/srep26765 PMC4886214

[78] B. Bettler , J. Y. H. Tiao , Pharmacol Ther 2006, 110, 533. 16644017 10.1016/j.pharmthera.2006.03.006

[79] J. Y. Kang , J. Chadchankar , T. N. Vien , M. I. Mighdoll , T. M. Hyde , R. J. Mather , T. Z. Deeb , M. N. Pangalos , N. J. Brandon , J. Dunlop , S. J. Moss , J. Biol. Chem. 2017, 292, 6621. 28213518 10.1074/jbc.M116.772541 PMC5399111

[80] C. Erbel‐Sieler , C. Dudley , Y. Zhou , X. Wu , S. J. Estill , T. Han , R. Diaz‐Arrastia , E. W. Brunskill , S. S. Potter , S. L. McKnight , Proc. Natl. Acad. Sci. U S A 2004, 101, 13648. 15347806 10.1073/pnas.0405310101 PMC518807

[81] Y. Hashimoto , T. Niikura , H. Tajima , T. Yasukawa , H. Sudo , Y. Ito , Y. Kita , M. Kawasumi , K. Kouyama , M. Doyu , G. Sobue , T. Koide , S. Tsuji , J. Lang , K. Kurokawa , I. Nishimoto , Proc. Natl. Acad. Sci. U S A 2001, 98, 6336. 11371646 10.1073/pnas.101133498 PMC33469

[82] B. Chen , C. Xie , T. Shi , S. Yue , W. Li , G. Huang , Y. Zhang , W. Liu , Neurobiol. Dis. 2023, 176, 105936. 36511337 10.1016/j.nbd.2022.105936

[83] J. Aruga , K. Mikoshiba , Mol. Cell. Neurosci. 2003, 24, 117. 14550773 10.1016/s1044-7431(03)00129-5

[84] N. Puranik , M. Song , Biomolecules 2024, 14, 1060. 39334827 10.3390/biom14091060 PMC11430182

[85] A. Carmignani , M. Battaglini , E. Sinibaldi , A. Marino , V. Vighetto , V. Cauda , G. Ciofani , ACS Appl. Nano Mater. 2022, 5, 1702.

[86] Y. Liu , K. Ai , L. Lu , Chem. Rev. 2014, 114, 5057. 24517847 10.1021/cr400407a

[87] X. Wang , J. Zhang , Y. Wang , C. Wang , J. Xiao , Q. Zhang , Y. Cheng , Biomaterials 2016, 81, 114. 26731575 10.1016/j.biomaterials.2015.11.037

[88] X. Zheng‐Bradley , I. Streeter , S. Fairley , D. Richardson , L. Clarke , P. Flicek , Gigascience 2017, 6, 1. 10.1093/gigascience/gix038 PMC5522380 28531267

[89] A. Dobin , C. A. Davis , F. Schlesinger , J. Drenkow , C. Zaleski , S. Jha , P. Batut , M. Chaisson , T. R. Gingeras , Bioinformatics 2013, 29, 15. 23104886 10.1093/bioinformatics/bts635 PMC3530905

[90] Y. Liao , G. K. Smyth , W. Shi , Bioinformatics 2014, 30, 923. 24227677 10.1093/bioinformatics/btt656

[91] Y. Liao , G. K. Smyth , W. Shi , Nucleic Acids Res. 2013, 41, e108. 23558742 10.1093/nar/gkt214 PMC3664803

[92] M. I. Love , W. Huber , S. Anders , Genome Biol. 2014, 15, 1. 10.1186/s13059-014-0550-8 PMC4302049 25516281

[93] Q. Ren , H. Ma , L. Wang , J. Qin , M. Tian , W. Zhang , Curr. Genomics 2024, 26, 24. 39911279 10.2174/0113892029298721240627095839 PMC11793068

[94] G. Yu , L. G. Wang , Y. Han , Q. Y. He , OMICS 2012, 16, 284. 22455463 10.1089/omi.2011.0118 PMC3339379

