# Supplementary material for: Polydopamine‐Based Antioxidant Countermeasures Against Spaceflight‐Induced Neurodegeneration
Source: Small Sci. 2025 Dec 15;6(1):e202500510. doi: 10.1002/smsc.202500510 (PMC12794679; doi:10.1002/smsc.202500510)
Supplement: Supplementary file 1 — Supplementary Material [file SMSC-6-e202500510-s001.pdf]

Polydopamine-Based Antioxidant Countermeasures against Spaceflight-Induced Neurodegeneration

Alessio Carmignani[a,*], *Attilio Marino*[a], *Matteo Battaglini*[a], *Melike Belenli Gümüş*[a], *Elisa Carrubba*[b], *Michele Balsamo*[b], *Giovanni Valentini*[c], *Gabriele Mascetti*[c], *Marco Vukich*[d], *Giada Graziana Genchi*[a,e,*,#], *Gianni Ciofani*[a,*,#]

[a]Istituto Italiano di Tecnologia, Smart Bio-Interfaces, Viale Rinaldo Piaggio 34, 56025 Pontedera, Italy

[b]Kayser Italia S.r.l., Via di Popogna 501, 57128 Livorno, Italy

[c]Agenzia Spaziale Italiana, Via del Politecnico snc, 00133 Roma, Italy

[d]ESA/ESTEC, HESpace, Keplerlaan 1, 2200 AG Noordwijk, The Netherlands

[e]University of Bari "Aldo Moro", Department of Bioscience, Biotechnology and Environment, Via Orabona 4, 70125 Bari, Italy

*Corresponding Authors: alessio.carmignani@iit.it; giada.genchi@iit.it; gianni.ciofani@iit.it

[#]Equally contributing Authors

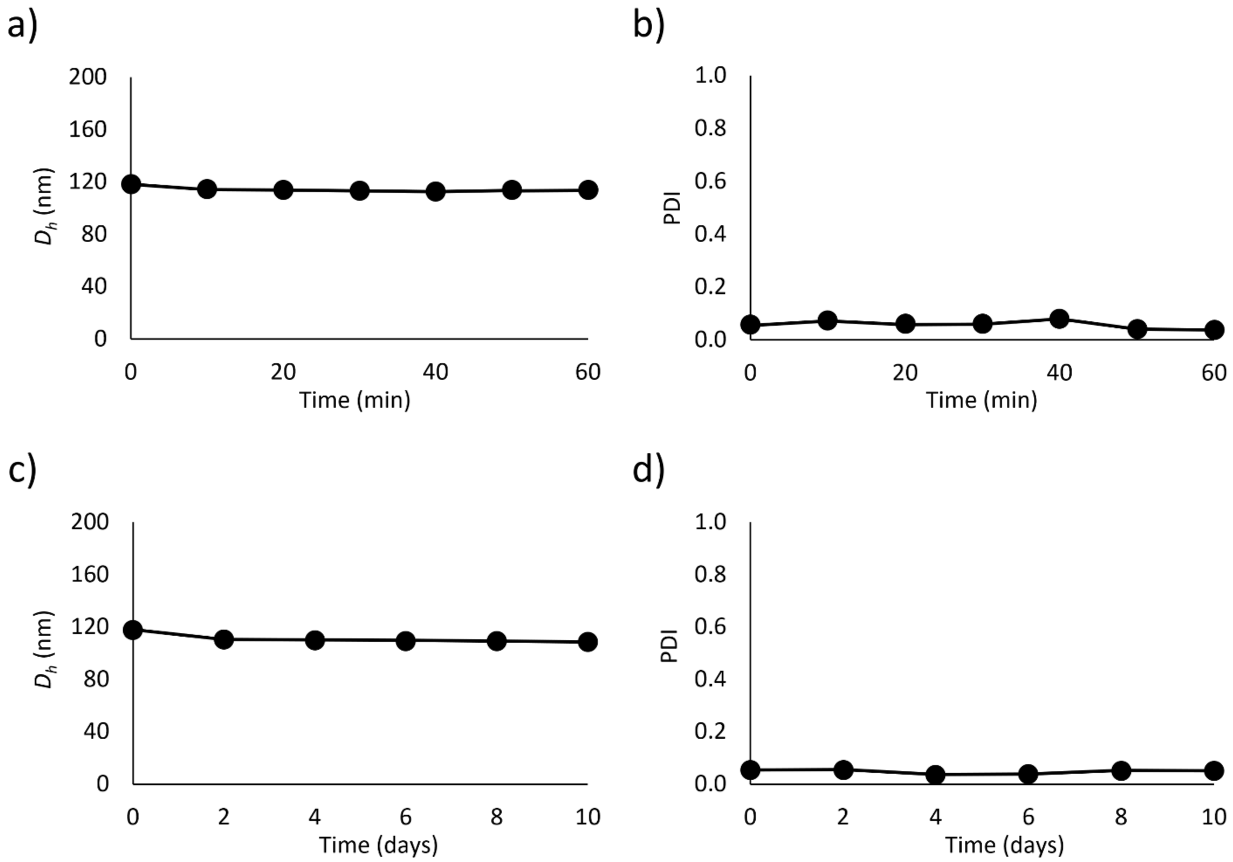

Figure S1. PDNP stability evaluation performed in differentiation medium. Analysis of a-c) D_h and b-d) PDI over a-b) 1 h and c-d) 10 days.

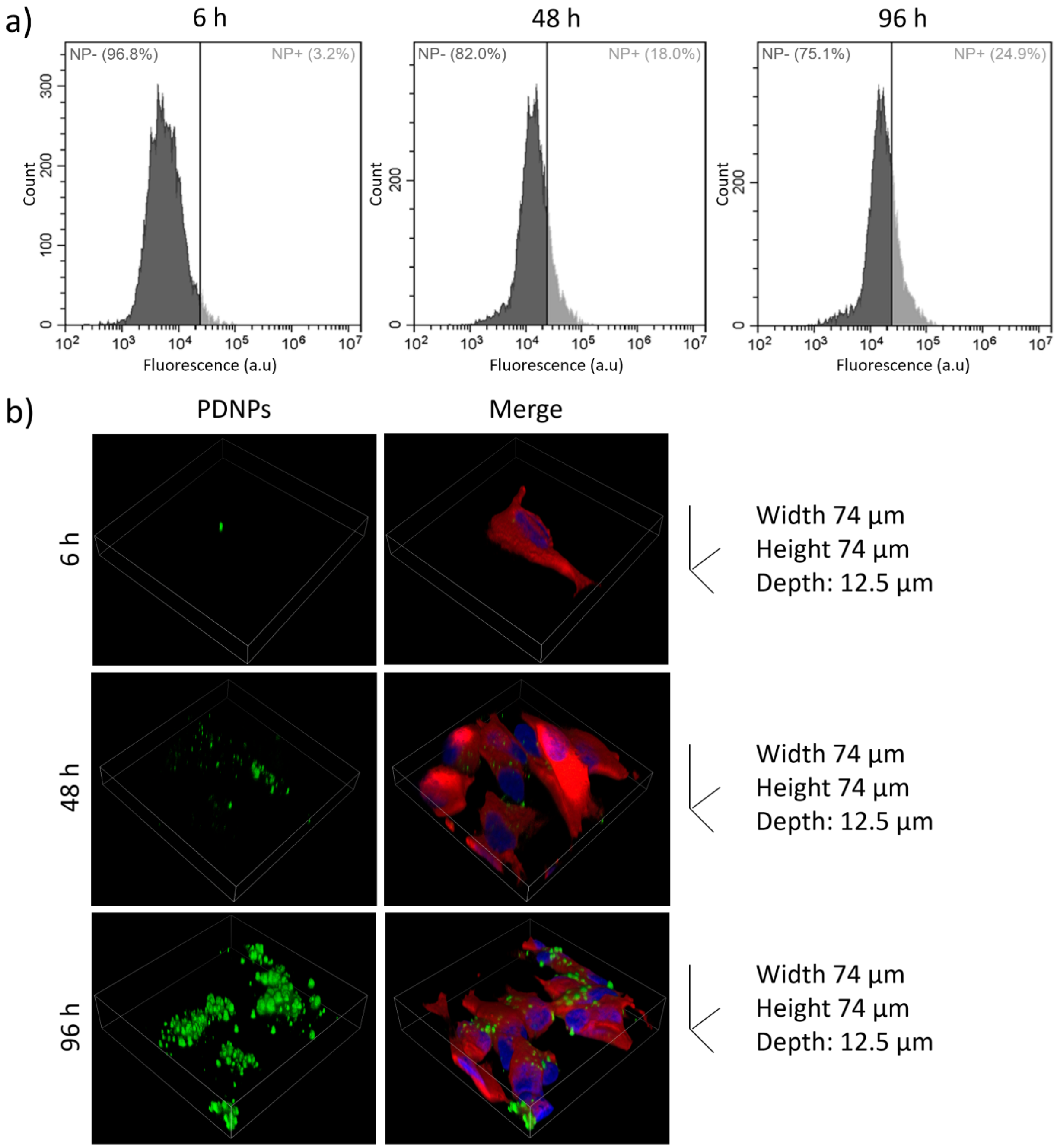

Figure S2. PDNP internalization assessment in Earth gravity. a) Representative flow cytometry plots (PDNP-negative celld in dark gray, PDNP-positive celld in light gray). b) Representative 3D confocal microscopy image reconstructions (f-actin in red, DiO-PDNPs in green, nuclei in blue).

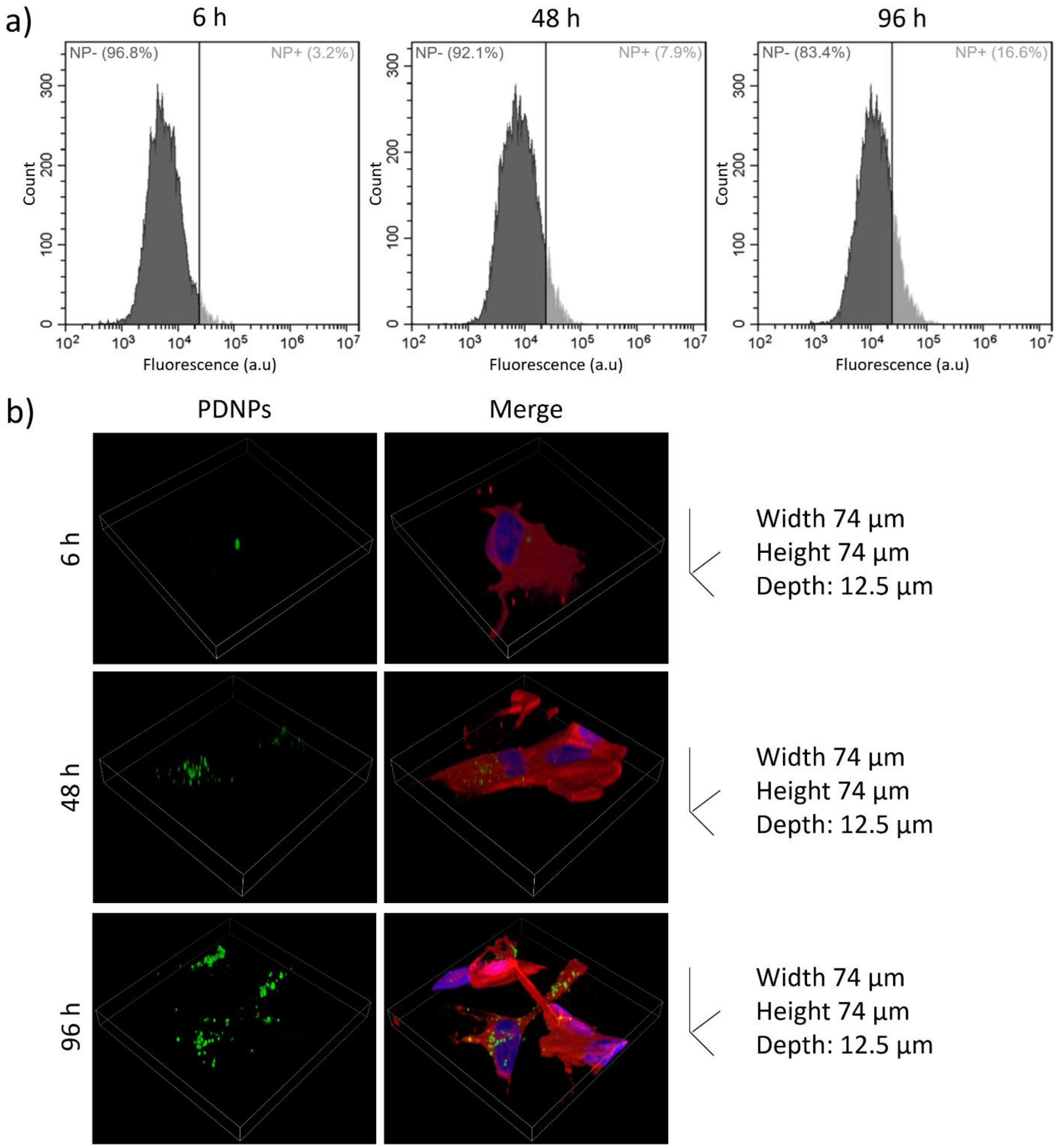

Figure S3. PDNP internalization assessment in simulated microgravity. a) Representative flow cytometry plots (PDNP-negative cells in dark gray, PDNP-positive cells in light gray). b) Representative 3D confocal microscopy image reconstructions (f-actin in red, DiO-PDNPs in green, nuclei in blue).

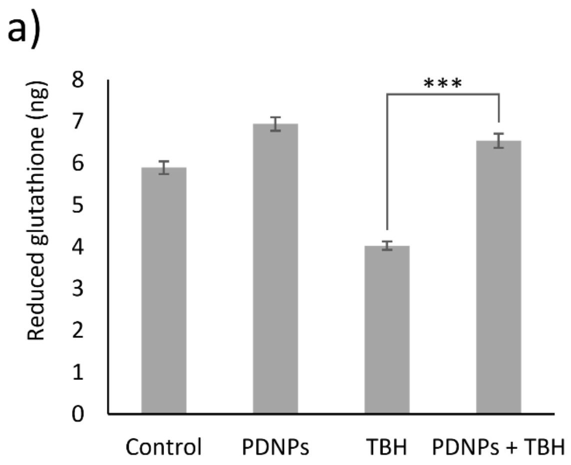

Figure S4. Reduced glutathione (GSH) levels assessment. Quantification of GSH levels in a) Earth gravity and b) simulated microgravity conditions (n = 3, *** p < 0.001).

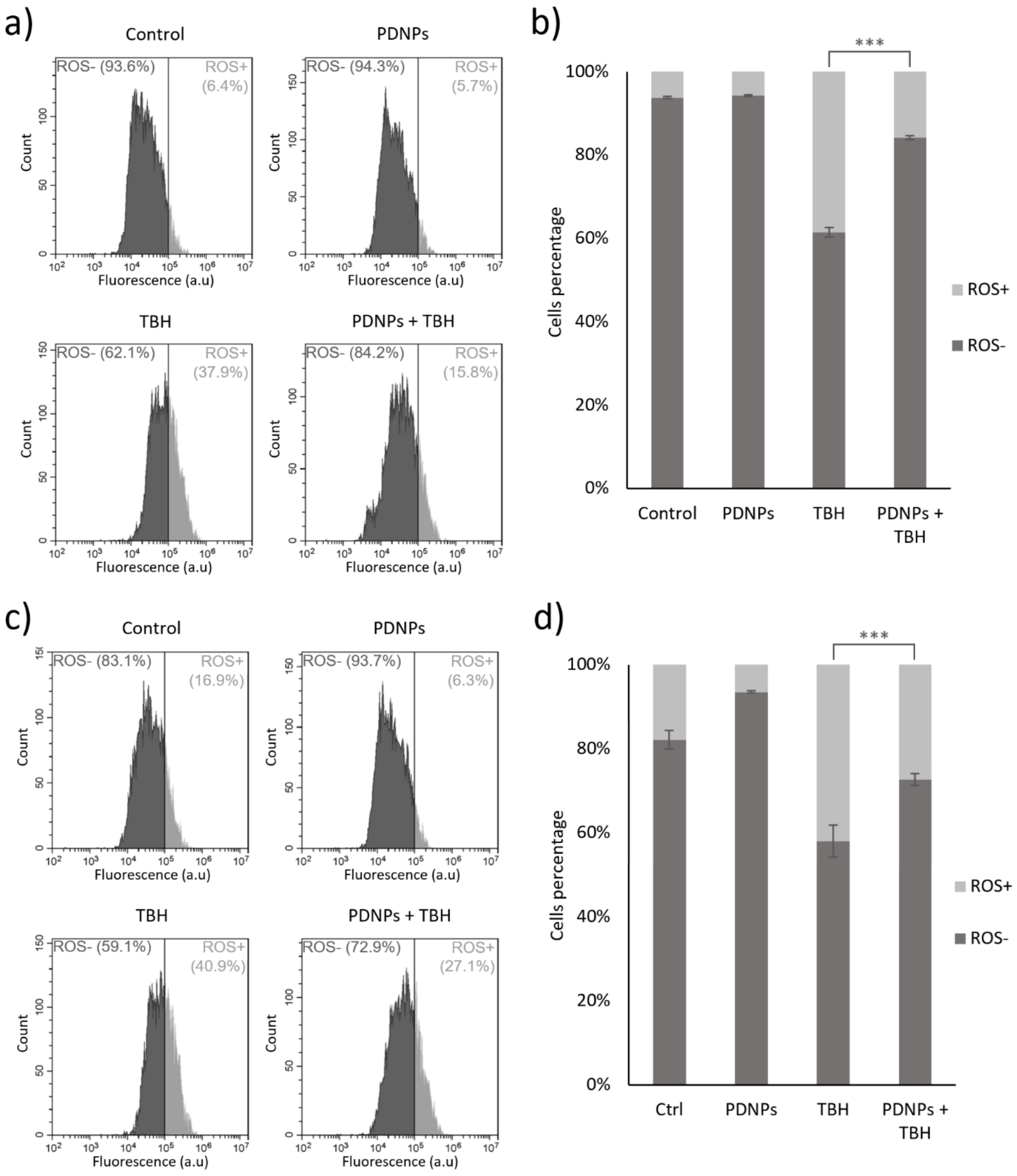

Figure S5. Intracellular oxidative stress level analysis. a) Representative plots and b) flow cytometry analysis for cultures in Earth gravity conditions (ROS-negative cell in dark gray, ROS-positive cell in light gray). c) Representative plots and d) flow cytometry analysis for cultures in simulated microgravity (ROS-negative cell in dark gray, ROS-positive cell in light gray; $n = 3$, *** $p < 0.001$).

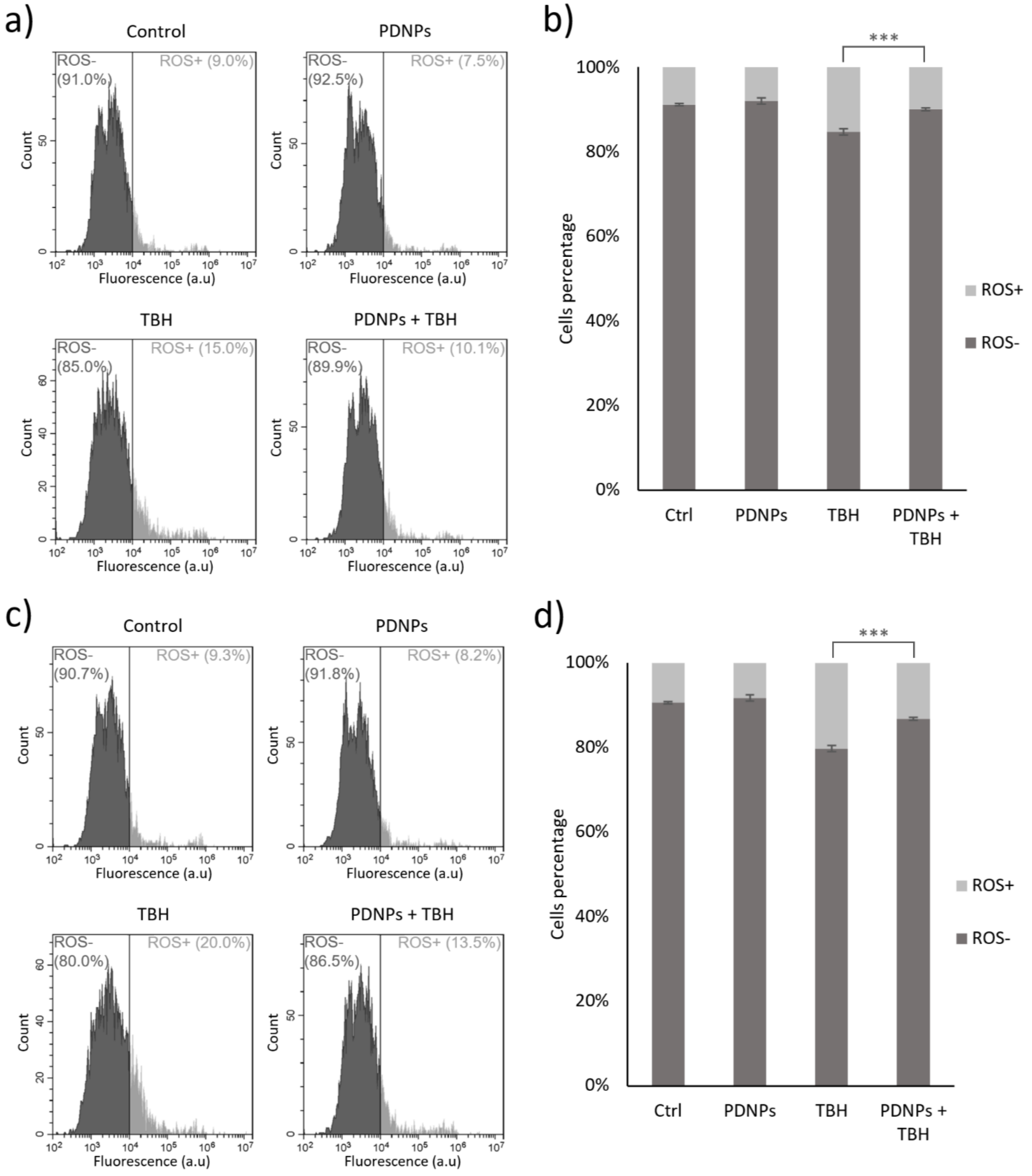

Figure S6. Mitochondrial oxidative stress level analysis. a) Representative plots and b) flow cytometry analysis for cultures in Earth gravity conditions (ROS-negative cell in dark gray, ROS-positive cell in light gray). c) Representative plots and d) flow cytometry analysis for cultures in simulated microgravity (ROS-negative cell in dark gray, ROS-positive cell in light gray; $n = 3$, *** $p < 0.001$).

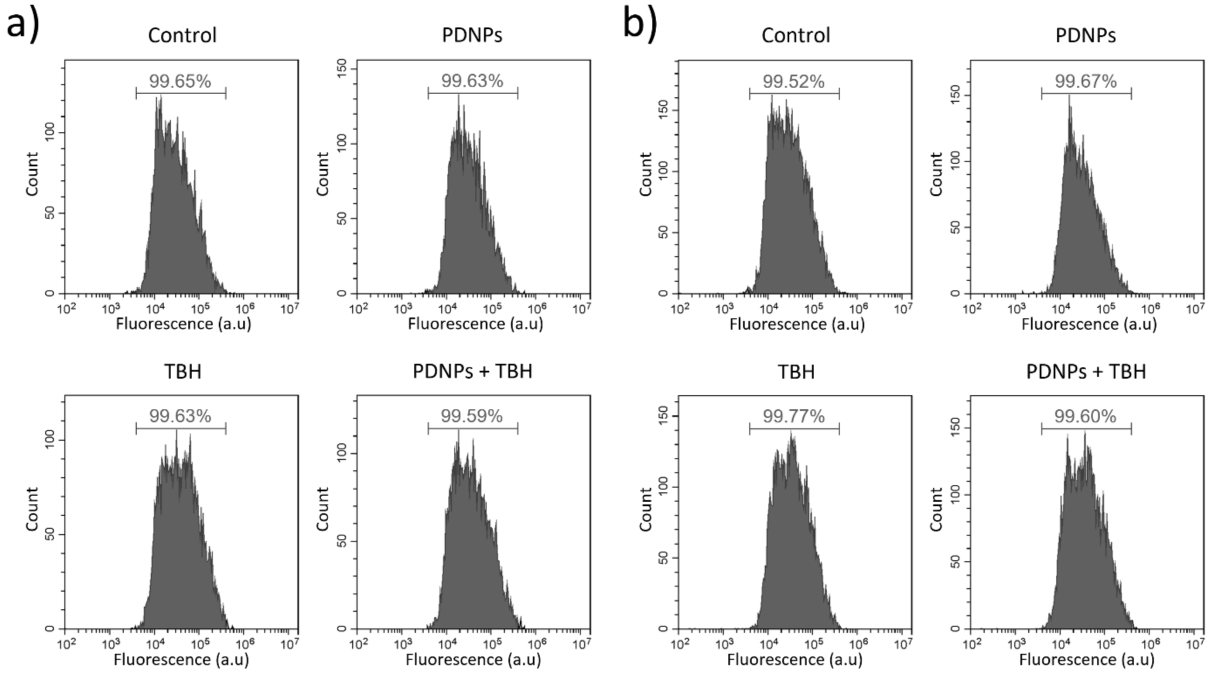

Figure S7. Mitochondrial membrane potential analysis. Representative flow cytometry plots for cultures in a) Earth gravity and b) simulated microgravity conditions.

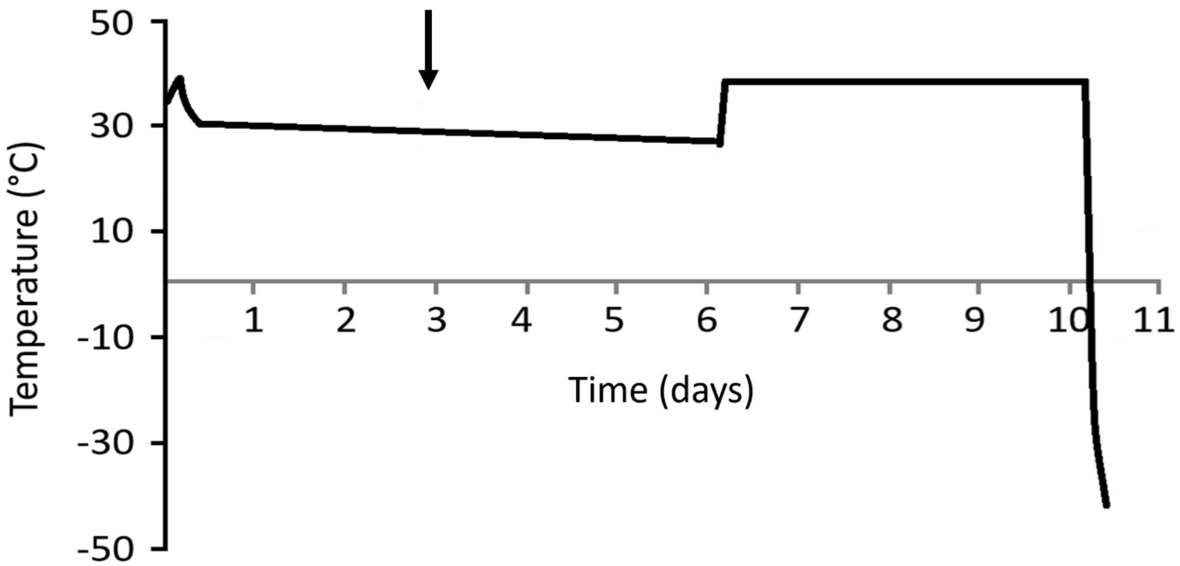

Figure S8. Thermal profile of the in-flight experiment. The arrow indicates payload launch time.

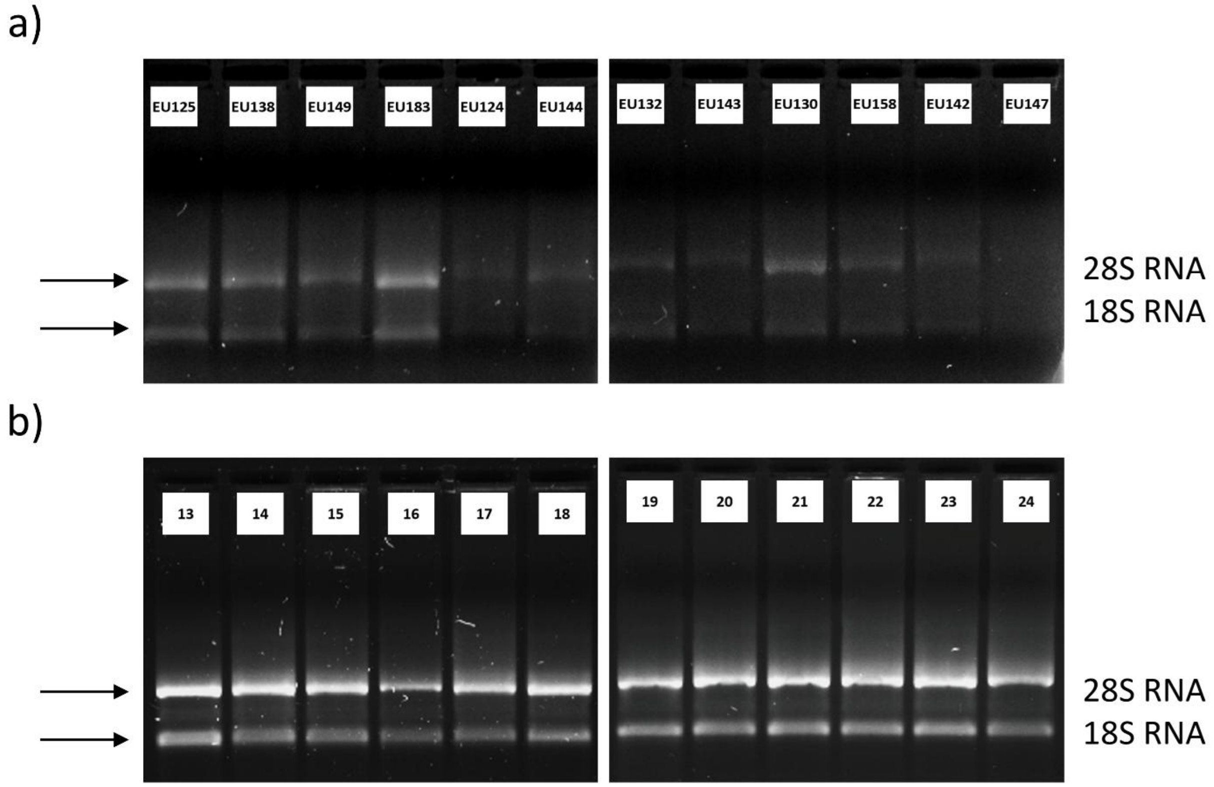

Figure S9. RNA integrity assessment. Agarose gel electrophoresis of extracted/purified RNA, respectively, from a) in-flight and b) ground control samples. The arrows indicate 28S and 18S ribosomal RNAs.

a)

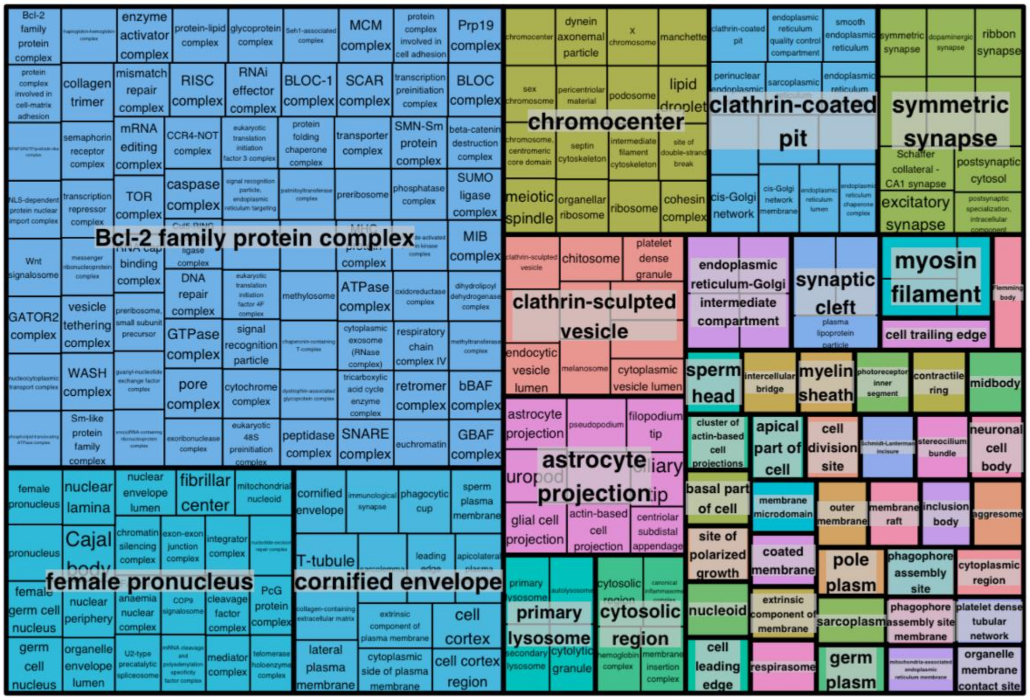

b)

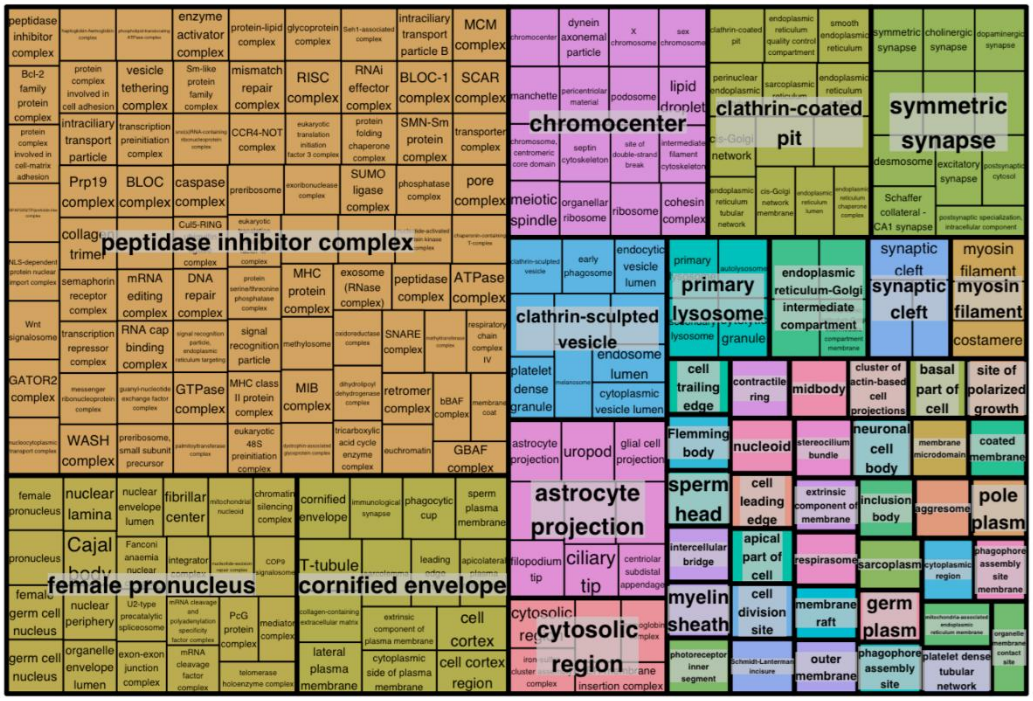

Figure S10. Gene ontology (GO) enrichment analysis. Treemaps visualization of redundant GO enriched terms resuming a) µ*g* and b) CR effects.

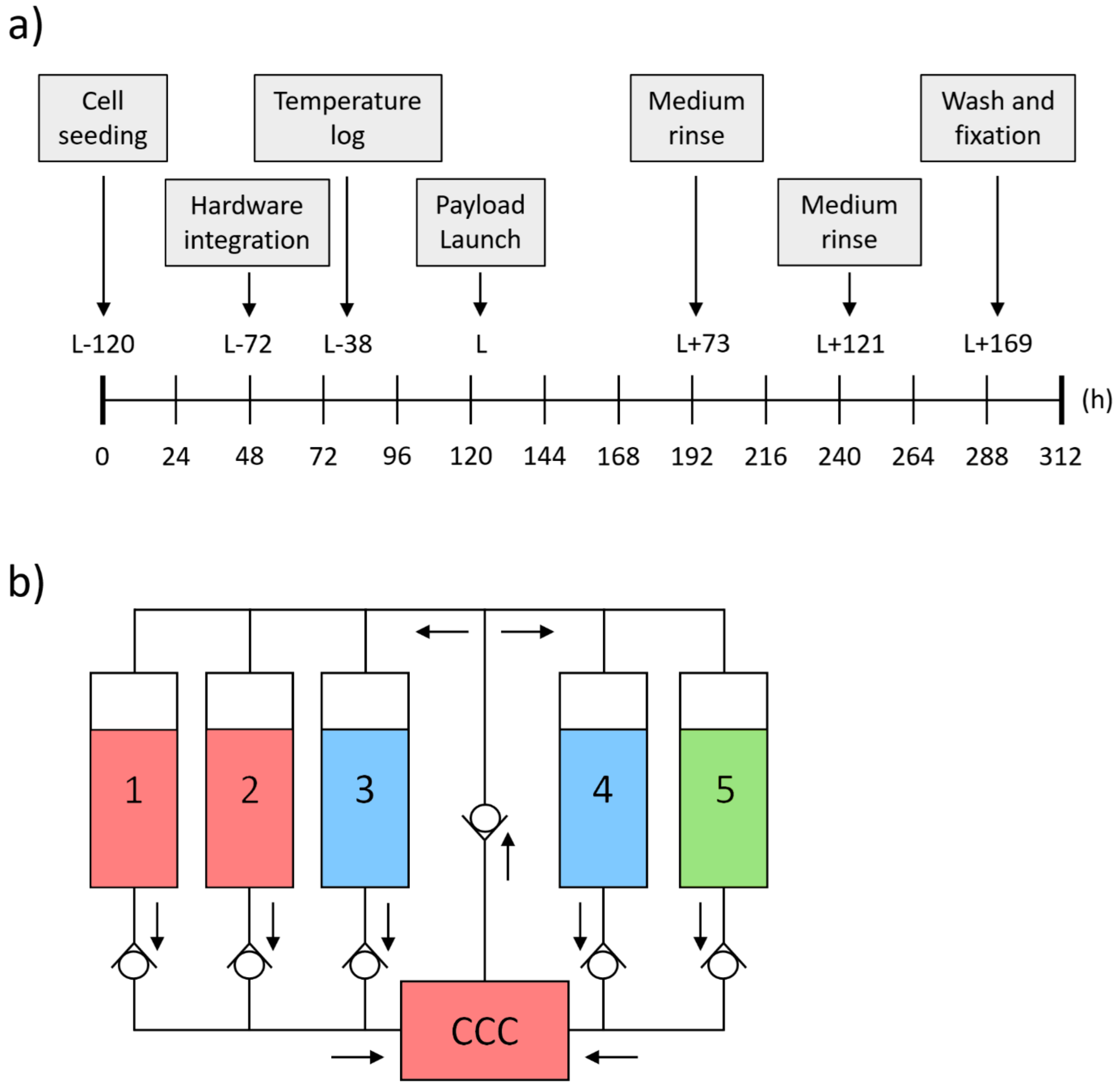

Figure S11. In-flight experiment. a) Experiment timeline with the indication of relevant time points (L = payload launch). b) Diagram of the experimental flow circuit, depicting the positions of the five reservoirs: 1,2 differentiation medium; 3, 4 DPBS; 5 fixative (CCC = cell culture chamber).

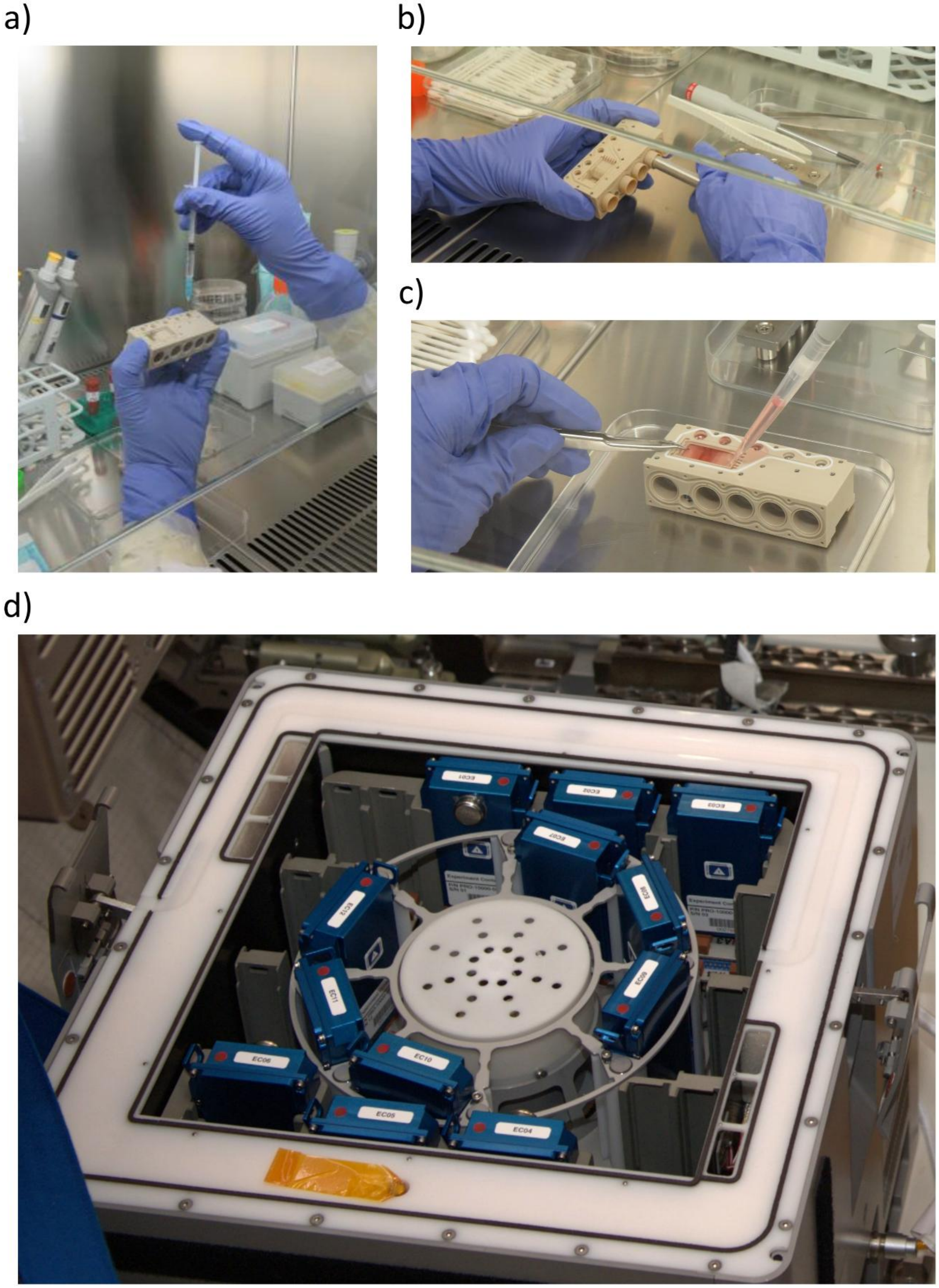

Figure S12. Experimental units (EUs) integration. a-c) Representative pictures and d) positioning inside the Kubik incubator/centrifuge.

Table S1. Comparative antioxidant performance of PDNPs and of reference compounds.

Compound	Concentration tested	Trolox equivalent (µM)
PDNPs	1 µg/mL	69.77 ± 0.60
Ascorbic acid	100 µM	78.25 ± 1.09
Tannic acid	10 µM	106.13 ± 0.94
Idebenone	100 µM	134.67 ± 2.11

Table S2. Summary of differential expression analysis for protein-coding genes.

Comparison	Upregulated genes	Downregulated genes	Total DEGs
H vs G	227	105	332
B vs A	215	1439	1654
B vs G	332	155	487
A vs G	295	1177	1472
A vs C	303	2535	2838
C vs G	978	2724	3702
F vs E	803	854	1657
B vs C	535	58	593
B vs E	637	1507	2144

Experimental classes: A (-PDNP, µ*g*, +CR); B (+PDNP, µ*g*, +CR); C (-PDNP, s1*g*, +CR); D (+PDNP, s1*g*, +CR); E (-PDNP, sµ*g*, -CR); F (+PDNP, sµ*g*, -CR); G (-PDNP, 1*g*, -CR); H (+PDNP, 1*g*, -CR).
